# Nucleic acid ligands act as a PAM and agonist depending on the intrinsic ligand binding state of P2RY2

**DOI:** 10.1073/pnas.2019497118

**Published:** 2021-04-28

**Authors:** Masaki Takahashi, Ryo Amano, Michiru Ozawa, Anna Martinez, Kazumasa Akita, Yoshikazu Nakamura

**Affiliations:** ^a^Project Division of RNA Medical Science, The Institute of Medical Science, The University of Tokyo, Tokyo 108-8639, Japan;; ^b^RIBOMIC Inc., Tokyo 108-0071, Japan

**Keywords:** GPCR, nucleic acid aptamer, PAM agonist, P2RY2

## Abstract

G protein–coupled receptors (GPCRs) play diverse roles in physiological processes, and hence the ligands to modulate GPCRs have served as important molecules in biological and pharmacological approaches. However, the exploration of novel ligands for GPCR still remains an arduous challenge. In this study, we report a method for the discovery of nucleic acid ligands against GPCRs by an advanced RNA aptamer screening technology that employs a virus-like particle (VLP), exposing the GPCR of interest. An array of biochemical analyses coupled with a cell-based assay revealed that one of the aptamers raised against purinergic receptor P2Y2 (P2RY2), a GPCR, exhibits an activation potency to unliganded receptor and prohibits a further receptor activation by endogenous ligand, behaving like a partial agonist. However, the aptamer enhances the activity of intrinsic ligand-binding P2RY2, thereby acting as a positive allosteric modulator (PAM) to liganded receptor. Our findings demonstrate that the nucleic acid aptamer conditionally exerts PAM and agonist effects on GPCRs, depending on their intrinsic ligand binding state. These results indicate the validity of our VLP-based aptamer screening targeting GPCR and reemphasize the great potential of nucleic acid ligands for exploring the GPCR activation mechanism and therapeutic applications.

## Significance

Discovery of ligands for G protein–coupled receptors (GPCRs) is of importance in receptor biology and pharmacology but is still a challenging issue. Here, we propose a method for the discovery of ligands against GPCRs by employing a virus-like particle (VLP) and show unique properties of identified nucleic acid aptamers for GPCR. One aptamer raised against purinergic receptor P2Y2 (P2RY2), a GPCR, behaves like a partial agonist to unliganded receptor, whereas it exhibits a positive allosteric modulator (PAM) activity to liganded receptor. We demonstrate the validity of our aptamer screening method targeting VLP-stabilized GPCR and a unique aptamer with dual function, agonist and PAM, for GPCR, depending on whether the intrinsic ligand is prebound to the receptor.

G protein–coupled receptors (GPCRs) are involved in varied human physiological functions and are targets for one-third of currently marketed drugs (1). Besides studies on specific inhibitors and activators interacting with the orthosteric ligand-binding pockets of GPCRs, recent studies shedding light on allosteric modulators have drawn attention for providing a deeper understanding of the molecular mechanisms underlying GPCR activation that may be exploited for potential therapeutics (2, 3). However, the technology for developing novel compounds targeting GPCRs has inherent technical hurdles for high expression and purification of GPCRs in their native conformation. An innovative platform remains to be developed to produce any type of GPCRs abundantly and easily while retaining the native conformation.

Nucleic acid aptamers are single-stranded oligonucleotides that adopt diverse conformations and recognize target epitopes with relatively high shape complementarity (4). Aptamers are isolated in vitro from combinatorial libraries composed of approximately a quadrillion molecules that give rise to a vast set of three-dimensional (3D) structures based on their primary sequence and chemistry. The first conceptual method is referred to as systematic evolution of ligands by exponential enrichment (SELEX) (5, 6) and has generated various experimental and pharmacological agents (7, 8). One of the most important features of SELEX (or aptamer) is that aptamers can be generated against various targets with high affinity and specificity, regardless of the molecular weight and complexity of targets, which range from small chemical compounds to recombinant proteins, bacteria, viruses, and cultured cells. Along with a wide range of target tolerances, technological advances in sequencing and bioinformatics have substantially expanded the potential of aptamer discovery (9–12). Nonetheless, few aptamers targeting cell-surface proteins, especially GPCRs, have been reported because of a poor integration of the technologies, despite advances in each relevant technique and expertise.

As aptamers specifically recognize targets with shape complementarity, it is vital to choose and prepare selection targets in their native conformation. Accordingly, although several types of selection materials targeting cell-surface proteins (e.g., recombinant proteins, membrane extract, and whole cells) have been used (13, 14), the most promising source material appear to be live whole cells, which ensure the native target structure even in case of unstable proteins. However, such SELEX imposes several restrictions on the selection process because the effects on cell viability must be considered for ensuring target structure and for reducing noise proteins arising from dead cells. Moreover, SELEX using cells and other crude materials concomitantly give rise to various aptamers bound to nontarget proteins. To address this, in addition to analyzing a huge number of sequences in enriched libraries, in silico analysis is required to unveil concealed aptamers bound to the target from numerous sequences. A recent study highlighted the importance of such technologies (e.g., high-throughput sequencing and bioinformatics) to isolate aptamers bound to a specific GPCR embedded in detergent micelles that mimic the lipid bilayer (15). However, this method is only applicable to certain GPCRs whose structure is highly stable, such as beta2-adrenoreceptor.

**Fig. 1.**
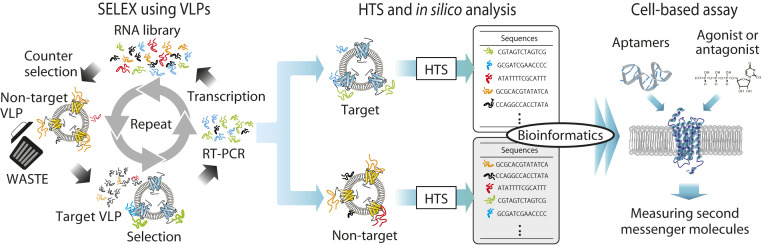
Schematic drawing of the SELEX strategy using VLPs. After selection with target VLP and counter selection with nontarget VLP, enriched sequences bound to the target and nontarget VLP at final round (seventh round) were examined by high-throughput sequencing (HTS) and in silico analysis, followed by cell-based assays.

Here, we propose a method for exploring nucleic acid aptamers as ligands for GPCRs by employing virus-like particles (VLPs) and immobilization-free partitioning manner (Fig. 1). Based on this SELEX method using VLPs (VLP-SELEX), we screened aptamers against the purinergic receptor P2Y2 (P2RY2) as a model target of GPCR and elucidated their functional properties. An array of biochemical analysis coupled with cell-based assay revealed that one of the aptamers shows dual function depending on whether the intrinsic ligand, uridine triphosphates (UTP), is bound to the receptor. When P2RY2 is unliganded, the aptamer has activation potency and prohibits further receptor activation by endogenous ligand UTP. In contrast, when P2RY2 is liganded, the aptamer has a positive allosteric modulator (PAM) activity to a UTP-bound receptor. These findings suggest that the aptamer binds to an allosteric site of the receptor and prevents UTP from activating an unliganded GPCR by occluding the orthosteric binding pocket and/or by changing the conformation of the binding pocket. To our knowledge, this is the first bifunctional aptamer with dual agonist and PAM activity for GPCR depending on whether endogenous ligand is prebound to the receptor. Our results reinforce the validity of VLP-SELEX targeting GPCR to discover aptamers for GPCRs, and the high potential of nucleic acid ligands for elucidating the mechanism(s) of GPCR activation and expanding therapeutic strategies.

## Results

### Aptamer Selection for GPCR.

To develop nucleic acid aptamers for GPCRs, we established an advanced SELEX technology by employing a VLP- exposing GPCR of interest (Fig. 1). We used a library composed of modified nucleotides with 2′-fluoro-pyrimidine, 2′-deoxy-adenosine and 2′-hydroxy-guanosine for tolerance to nucleases because VLPs targeted in SELEX are relatively crude material. The human P2RY2 was used as a model GPCR because the molecular basis of P2RY2 activation remains unknown, despite its relevance to many pathophysiological states (16–19). Using a commercially available system, a VLP-expressing P2RY2 was produced and then the receptor expression was confirmed (*SI Appendix*, Fig. S1). During the SELEX cycle (*SI Appendix*, Table S1), we used an immobilization-free separation system with an ultrafiltration column capable of separating aptamers complexed with VLPs from free RNA sequences to avoid any conformational distortion of GPCR induced by immobilization (*SI Appendix*, Fig. S2). To remove noise sequences, counterselection was performed with a VLP-expressing endothelin B receptor (EDNRB, as a nontarget GPCR), similar to the previous study (20). At the final selection (round 7), the enriched library was mixed with the target and nontarget VLPs, and sequences bound to each VLP were examined by high-throughput sequencing (HTS) as positive and false-positive sequence data, respectively. Then, the sequence data were further analyzed using FASTAptamer (21) to efficiently discover potential sequences based on the read per million (RPM) and enrichment (ratio of RPM of sequences in positive to that in false-positive sequence data) (details in *Materials and Methods* and Dataset S1).

**Fig. 2.**
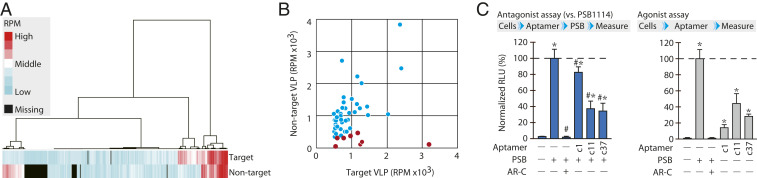
Selection of candidate aptamers from HTS data. (*A*) Heatmap analysis. The difference in the read number (RPM) of each aptamer, composing the enriched library to VLPs expressing target GPCR (P2RY2) and nontarget GPCR (EDNRB), was visualized by a heatmap hierarchical clustering. (*B*) Scatter plot from HTS data. Based on the RPM of the data from sequences bound to the target VLP (x-axis) and that from sequences bound to the nontarget VLP (y-axis), 51 enriched sequences representing each cluster were shown. The eight sequences that were enriched more than twofold in target-associated sequences compared to nontarget-associated sequences are shown in red. Each dot represents the sequences showing the highest RPM in each cluster. (*C*) Effects of aptamers on the P2RY2 activation. Inhibitory potency of the aptamers (c1, c11, and c37, final 2.5 μM) and chemical antagonist AR-C118925XX (AR-C, final 10 μM) were examined when the cells overexpressing P2RY2 were posttreated by chemical agonist PSB1114 (PSB, final 100 nM) (*Left*). In the agonist assay (*Right*), the cells overexpressing P2RY2 were treated by aptamers (final 5 μM) and PSB (final 100 nM). The pretreatment of AR-C at a concentration of 10 μM was performed to confirm the PSB specificity. Data represent the mean ± SD (n = 3 independent experiments). The values were expressed as relative luminescent units (RLU shown in %) to the 100 nM PSB1114 level without aptamer after subtraction of basal LU in control cells without treatment. ^#^*P* < 0.05 versus PSB1114 treatment group without aptamer in the antagonist assay; and *P < 0.05 versus no treatment group in both assays.

A subsequent heatmap analysis clearly visualized the difference in the read number of each aptamer, composing the enriched library between the target and nontarget VLPs (Fig. 2*A*). Furthermore, a scatter plot based on the above-mentioned clustering analysis with FASTAptamer indicated eight enriched sequences with the highest RPM in each of the top eight clusters (see *Materials and Methods* and Fig. 2*B*). These eight sequences were then subjected to motif and cluster analyses (*SI Appendix*, Fig. S3). The in silico analyses highlighted the sequences c1, c11, and c37 that represent unique compositions of defined motifs, which were subjected to further functional analyses.

### Functional Assessment of Isolated Aptamers.

Since agonist-mediated P2RY2 activations evokes calcium signaling via the Gαq protein (22), the effect of aptamer on P2RY2 function was examined by measuring the second messenger, calcium ions, using an Aequorin-based assay. Pretreatment of P2RY2-overexpressing HEK293 cells with each aptamer inhibited the calcium signaling induced by the specific chemical agonist PSB1114, thereby indicating the inhibitory effect of the aptamers, particularly in c11 and c37, on the molecular interaction between the receptor and chemical agonist PSB1114 (Fig. 2*C* and *SI Appendix*, Fig. S4). Nevertheless, to our surprise, c11 and c37 exerted an activation potency on P2RY2 when PSB1114 was absent (Fig. 2*C* and *SI Appendix*, Fig. S5). In terms of specificity, the aptamer-induced activation to P2RY2 disappeared in cells pretreated with the specific antagonist AR-C118925XX and in cells without introducing the P2RY2-expression vector (*SI Appendix*, Fig. S6). Furthermore, the treatment by aptamers c11 and c37 showed no activity toward the P2Y receptor family proteins P2RY4 and P2RY11, despite both receptors being activated by the same intrinsic agonists as P2RY2 (*SI Appendix*, Figs. S7 and S8). These findings demonstrate that c11 and c37 target the exogenously expressed P2RY2, reinforcing the target engagement.

### Estimation of Critical Region Harnessing Activation Potency to Receptor.

**Fig. 3.**
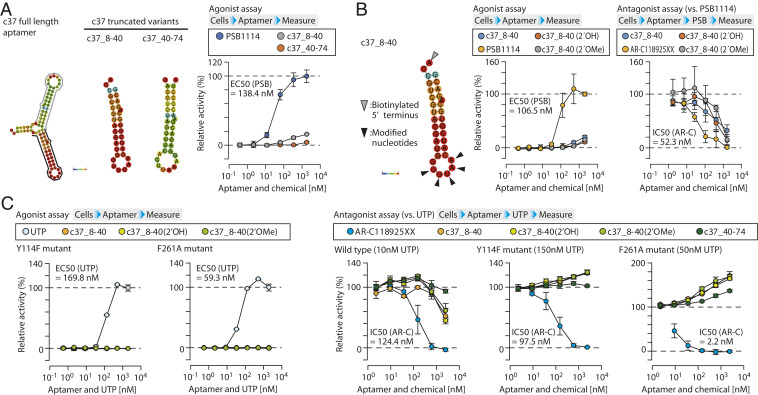
Effect of chemical modification of the aptamers on their activity. (*A*) Estimation of critical region possessing activation potency. The predicted secondary structure of the c37 aptamer and its truncated variants were shown (*Left*). Activation potency of the truncated aptamers was examined. The values were expressed as RLU to the 2.5 μM PSB1114 level without aptamer (*Right*). (*B*) Effect of the chemical modification of aptamer. The predicted secondary structure of the aptamer c37_8-40 is shown (*Left*). The unpaired 2′-fluoro-uridine and 2′-deoxy-adenosine located in the loop structure of c37_8-40, denoted by arrowheads, was modified with a hydroxy (2′OH) and methoxy group (2′OMe), respectively. Activation potency of the modified aptamers was measured and expressed as RLU to the 2.5 μM PSB1114 level (*Middle*). Inhibitory potency of the aptamers against 100 nM PSB1114 was expressed as RLU to the 100 nM PSB1114 level without aptamer as in *A* (*Right*). (*C*) Agonist and antagonist assay of aptamers to the mutant receptors. Activation potency of the modified aptamers to Y114F and F261A mutant receptors was shown as RLU to the 2.5 μM UTP (*Left*). Inhibitory activity of the indicated aptamers to each type of P2RY2 stimulated by UTP at the indicated concentrations was expressed as RLU to the indicated concentrations of UTP without aptamer in each type of P2RY2 (*Right*). Data represent the mean ± SD (n = 3 independent experiments). EC_50_ and IC50 values obtained from chemical agonists and antagonist were indicated.

To further investigate the biochemical properties of the newly identified aptamers in modulating receptor activation, we first determined the region conferring activation potency in each of the full-length c11 and c37 sequences. Based on the predicted secondary structure, the entire sequence of each aptamer was split into two stem-loop structures, giving rise to four truncates c11_8-39, c11_37-75, c37_8-40, and c37_40-74 (Fig. 3*A* and *SI Appendix*, Fig. S9). In an antagonist assay, each truncated sequence showed an inhibitory effect on PSB1114-induced calcium signaling in a dose-dependent manner (*SI Appendix*, Fig. S10*A*). In contrast, truncated aptamers c11_8-39 and c37_8-40 exhibited activation ability, whereas c11_37-75 and c37_40-74 were weak or nearly inactive (Fig. 3*A* and *SI Appendix*, Figs. S10*B* and S11*A*). These results suggest that the truncated sequences c11_8-39 and c37_8-40 possess the critical sequences that impart the functional potency to each full-length sequence. On the other hand, because there is no apparent difference in binding affinity between aptamers c37_7-40 and c37_40-74 (*SI Appendix*, Fig. S12), the difference in functional activities between the aptamers appears to depend on sequence-based functions rather than their binding affinities to the receptor. Thus, the truncated aptamer c37_8-40 showing relatively high activation and inhibitory potency to P2RY2 was investigated further by biochemical and cell-based assays.

### Effect of Chemical Modification on Activation Potency of Aptamer.

After estimating sequences conferring the unique aptamer activity, we then investigated the reasons underlying the activation ability of c37_8-40 to the receptor. It is well known that UTP and adenosine triphosphates (ATP) are intrinsic agonists for P2RY2 (19, 22), and their agonist potencies are drastically affected by chemical modifications of the ribose 2′ position (e.g., 2′-fluoro-UTP, 2′-deoxy-UTP, and 2′-O-methyl-UTP activities were reduced to 6%, 4%, and 0.3% of the 2′-hydroxy-UTP level, respectively) (19). Based on this report and the predicted secondary structure of aptamer c37_8-40, we examined whether unpaired nucleotides in the aptamer act as an agonistic moiety by modifying the 2′ position of the ribose in unpaired adenosine and uridine at the loop structure of the aptamer. As shown in Fig. 3*B* and *SI Appendix*, Fig. S11, the 2′ modifications in the truncated aptamer affected neither activation nor inhibitory potency, suggesting that unpaired nucleotides in the truncated aptamer do not activate the receptor via direct interaction with the ligand binding pocket.

### Activation Potency of Aptamer to Mutant Receptors.

To gain further insights into the mechanism underlying the activation potency of the truncated aptamer c37_8-40, its effects were evaluated on mutant receptors Y114F and F261A. It is noteworthy that Y114F and F261A substitutions in P2RY2 are located at the putative deep ligand-binding pocket and decrease calcium signaling evoked by the endogenous agonist UTP (23). The agonist assay with Y114F and F261A mutants indicated that the aptamer was inactive against both mutants (Fig. 3*C* and *SI Appendix*, Figs. S11 and S13). However, in the antagonist assay, the exposure of both mutant receptor-expressing cells to UTP after aptamer treatment resulted in increased calcium signaling in a dose-dependent manner, whereas the response of wild-type receptor to UTP was attenuated by pretreatment with the aptamer (Fig. 3*C* and *SI Appendix*, Fig. S11), as in the antagonist assay with the chemical agonist PSB1114 (*SI Appendix*, Fig. S4). These findings strongly suggest that c37_8-40 functions as a PAM to P2RY2, at least to Y114F and F261A mutants. Prior binding of c37_8-40 to the receptor still allows UTP to bind to the ligand pocket of the Y114F and F261A mutants, giving rise to enhanced calcium signaling, but not to the wild-type receptor, resulting in reduced calcium signaling. Given that both Y114F and F261A mutations are just single amino acid substitutions, the wild-type P2RY2 is also likely to have vacant space in the ligand binding pocket after aptamer binding. Moreover, because the chemical modification of the aptamer shown in Fig. 3*C* did not even affect PAM activity on the mutants, the unpaired nucleotides in the aptamer seemed not to interact with the deep orthosteric site of P2RY2 with or without the mutations.

### PAM Potency of Aptamer.

**Fig. 4.**
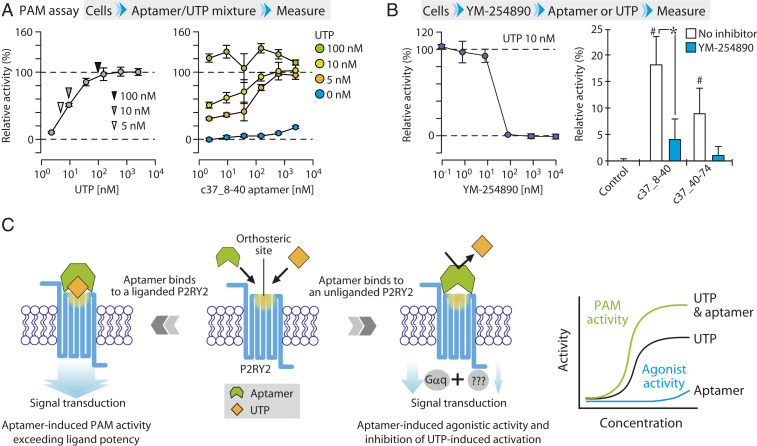
The predicated functions of isolated nucleic acid ligand. (*A*) Effect of simultaneous treatment with aptamer and UTP on wild-type P2RY2 activity. The dose–response curve using UTP is shown (*Left*). Cells overexpressing wild-type P2RY2 were exposed to the mixture of aptamer c37_8-40 and UTP at the indicated concentrations (*Right*). The values are expressed as RLU (in %) to the 2.5 μM (*Left*) and the 100 nM (*Right*) UTP level without aptamer. Data represent the mean ± SD (n = 3 independent experiments). (*B*) Effect of Gαq inhibitor on aptamer-induced calcium signaling. The effect of YM-254890 on the calcium signaling stimulated by 10 nM UTP was shown as RLU (in %) to the 10 nM UTP level without inhibitor (*Left*). Likewise, inhibitory effect of YM-254890 at 100 nM, which is sufficient to block calcium signaling activated by 10 nM UTP, was examined for the calcium signaling stimulated by 2.5 μM aptamers. The values are expressed as RLU (in %) to the 10 nM UTP level without aptamer. Control group is not exposed to the aptamers and inhibitor. Data represent the mean ± SD (n = 3 independent experiments). ^#^*P* < 0.05 versus control group; and *P < 0.05 between indicated groups. (*C*) Schematic drawing of the predicted functions of the aptamer. Our results suggest that c37_8-40 aptamer binds to an allosteric site of P2RY2 and exhibits dual function, agonist, and PAM, depending on whether the ligand UTP is prebound to the receptor.

Based on the above results, we hypothesized that the truncated aptamer c37_8-40 does not occupy the P2RY2 ligand pocket but rather prevents UTP from activating the receptor by covering the orthosteric site and/or by changing conformation of the ligand binding pocket in wild-type P2RY2. To test this hypothesis, the aptamer and UTP were simultaneously added to cells expressing a wild-type receptor (Fig. 4*A* and *SI Appendix*, Table S2). Unlike sequential exposure to UTP and the aptamer, exposure of cells to UTP-aptamer mixtures enhanced calcium signaling beyond the maximum response to endogenous agonist UTP. These results appear to support our hypothesis that the aptamer c37_8-40 binds to allosteric site(s) without occupying the UTP-interacting orthosteric site.

To gain more insights into the activation potency of c37_8-40 via allosteric site of P2RY2, an assay using YM-254890, a specific inhibitor for Gαq signaling, was performed (Fig. 4*B*). In this assay, we found that YM-254890 at 100 nM, which is sufficient to suppress calcium signaling activated by 10 nM UTP, could not fully inhibit the signaling transduction, although statistical significance was not observed between the control group without any treatment and the aptamer-treated group with inhibitor. Thus, it is speculated that noncanonical mediator(s) as well as Gαq protein are involved in the activation potency of aptamers mediated by the allosteric site.

Collectively, these findings demonstrate that the isolated nucleic acid aptamers exert an activation potency to an unliganded P2RY2, while inhibiting a further receptor activation by ligand UTP, and that the aptamer c37_8-40 enhances the affinity and/or efficacy of orthosteric agonists to a liganded P2RY2. Thus, these findings support the identification of a unique regulatory aptamer with conditional bifunctionality of being PAM and agonist, for purinergic GPCR, P2RY2 (Fig. 4*C*).

## Discussion

In this study, we present a unique nucleic acid ligand for GPCR and the validation of the aptamer screening strategy targeting GPCR by using VLP. A series of biochemical and cell-based analyses indicated that truncated c37_8-40 binds to an allosteric site and conditionally exhibits agonist and PAM functions depending on whether the ligand UTP is prebound to the receptor (Fig. 4*C*). Although the action mechanism remains unclear how the nucleic acid ligand inhibits a receptor activation by its endogenous ligand, to our knowledge, this is first report of a bifunctional aptamer with both PAM and agonist activity for GPCR, depending on the ligand binding state of the receptor.

In terms of the binding manner of the aptamer, our prediction seems reasonable considering the previous studies (23, 24). Although the crystal structure of P2RY2 remains to be solved, the studies using mutagenesis and computational approaches have proposed an agonist-binding model of P2RY2 where the base moiety of UTP interacts with a relatively deep area of the ligand-binding pocket. Furthermore, the allosteric site is predicted to be present in the shallow upper region of the pocket in close proximity to the orthosteric site. Our results showed that the chemical modification of unpaired nucleotides in the loop of the aptamer affected neither the activation nor the inhibitory potency of c37_8-40 (Fig. 3*B*). Furthermore, the 5′ and 3′ termini in c37_8-40 are adenosine modified by biotin, and guanosine, respectively, which appears to be inactive to P2RY2. Furthermore, a control experiment, using random library, reinforced that such activity of the aptamer is not nonspecific actions to the purinergic receptor P2Y2 as nucleic acid molecules (*SI Appendix*, Fig. S14). Based on these findings, the base moiety of unpaired nucleotides in the loop motif and termini of the aptamer is unlikely to activate the receptor by binding to the deep ligand binding pocket of P2RY2 but may interact with the putative allosteric site. Thus, we speculated that the aptamer c37_8-40 binds to the allosteric site without interfering with the orthosteric site as a PAM agonist for P2RY2. The reason why such a molecule was isolated from this selection is not obvious, but the presence of UTP and ATP in the culture medium and/or the propagation of a tiny amount of modified ribonucleoside triphosphates (rNTPs) from enzymatic step of aptamer synthesis (*SI Appendix*, Fig. S15) might contribute to a biased selection for generating aptamers that bind to the site(s) other than the orthosteric site.

To fully elucidate the unique properties of isolated aptamers, further studies are warranted. Nevertheless, our preliminary experiments raise an interesting possibility that the aptamer c37_8-40 induces the calcium signaling via noncanonical mediator(s) as well as Gαq proteins, since the aptamer-induced calcium signaling could not be completely suppressed by the specific Gαq inhibitor, YM 254890 (Fig. 4*B*). This might give us a clue to understand an unknown atypical signal transduction system of P2RY2 in the future. Moreover, when comparing aptamers c37_8-40 and c37_40-74, the aptamer c37_8-40 exhibited stronger activity as a PAM as well as an agonist (Fig. 3 *A* and *C*). Thus, further fundamental studies on conformation changes induced by these aptamers should be conducted to gain valuable insights into the structural bases for allosteric regulation of GPCR.

Owing to the limited ectodomains of GPCR as epitopes and infeasibility to retain their native conformation without a lipid bilayer, the generation of molecules that recognize the native GPCR conformation has been challenging. A few prior studies on GPCR used recombinant GPCR protein, such as NTS-1 (25) and beta2 adrenoreceptor (15), which can be overexpressed, purified, and reconstituted in native conformations. To expand the range of targeted GPCRs, our strategy harnesses a VLP that is readily available and is expected to enable the abundant expression of native conformation GPCRs, even for proteins with an unstable structure. In addition, expression of GPCRs in VLPs allows the GPCR purification process, a major obstacle, to be bypassed. Thus, by integrating VLPs and other available systems, our platform provides a robust and versatile approach for the discovery of a GPCR binding aptamers. Nonetheless, as this study is just the first trial targeting a single GPCR, P2RY2, further studies are required to validate versatility of our platform against a broader array of GPCRs. As the limitations of the current method, we cannot regulate the quaternary organization of GPCR, such as monomer, dimer, or higher-order oligomer. In addition, although VLP as a selection material can bypass a GPCR purification process, such crude materials are generally unsuitable for structural analysis and conventional molecular interaction assessment based on the surface plasmon resonance system because they contain a lot of noise (nontarget) proteins. For that reason, the present study only showed the rough estimation of dissociation constant values of the aptamers with flow cytometry using P2RY2 overexpressing cells (*SI Appendix*, Fig. S12). In addition to this caution about the purity of VLP, there is currently no convenient method to confirm the functions and quality of GPCRs on VLP and VLP itself; hence, it should be noted that there are not only advantages but also specific concerns in the usage of GPCR-stabilizing VLPs as a selection material for SELEX. We expect that GPCR-ligand discovery strategies based on nucleic acid aptamers will be further improved by adopting various technologies, such as a technique for single-cell sequencing analysis (26), structure-based in silico analysis (27), and GPCR-stabilizing techniques using detergent micelle (15) and nanodisc (28).

In summary, the present study provides a unique PAM agonist aptamer against P2RY2 and reemphasizes the high potential of an aptamer-driven ligand discovery strategy. VLP-SELEX presented here provides a feasible approach for exploring ligands bound to unstable cell-surface proteins like GPCRs. This strategy can be integrated with commercially and freely available materials and methods, and thus, has broad applicability with potential to change the current framework of GPCR-ligand discovery, uncovering the molecular basis underlying GPCR activation, and promoting drug discovery.

## Materials and Methods

### RNA and DNA Oligonucleotides.

DNA and RNA oligonucleotides used in this study were synthesized by and purchased from FASMAC Co. Ltd and GeneDesign, Inc., respectively.

### Chemicals.

PSB1114, AR-C118925XX, and YM-254890 were purchased from Tocris Bioscience. UTP and ribonucleoside triphosphate ATP (rATP) were purchased from GE Healthcare.

### Cell Culture and Transfection.

HEK293 cells were grown at 37 °C in Dulbecco's Modified Eagle's Medium (DMEM) (Wako Pure Chemical Industries), supplemented with 10% fetal bovine serum (FBS) (LifeTechnologies), 100 U/mL penicillin, and 100 μg/mL streptomycin (Wako) in a 5% CO_2_-humidified chamber. To express GPCR of interest in cultured cells, plasmid DNA encoding the GPCRs were transfected into HEK293 cells using polyethyleneimine.

### Production of VLP.

VLPs were produced by using the Expi293 Expression System (LifeTechnologies) according to the manufacturer's instructions. Briefly, VLP produced by a 37 mL culture medium were collected as a pellet and resuspended in 500 μL PBS. The suspension was divided into aliquots and stocked at −80 °C until use.

### Western Blotting.

Expression of transfected GPCRs in HEK293 cells and in VLP were examined by Western blotting. Cultured cells were lysed with radioimmunoprecipitation assay (RIPA) buffer containing 1x protease inhibitor mixture (Protease Inhibitor Mixture Tablets; Roche Diagnostics), and the protein concentration of the cell lysate was quantified with a Protein Quantification kit (Dojindo Molecular Technologies) according to the manufacturer’s instructions. Equal amounts of protein (∼20 μg) in the case of cell lysates or equal volumes of samples in the case of PBS-suspended VLPs were mixed with 4x sample buffer (0.25 M Tris HCl, 40% glycerol, 8% sodium dodecyl sulfate (SDS), 0.04% bromophenol blue, and 8% β-mercaptoethanol) without boiling and then separated by SDS-polyacrylamide gel electrophoresis (PAGE) with a 10% gel. After electrophoresis and blotting onto polyvinylidene fluoride (PVDF) membranes (Immobilon P; Millipore), the overexpressed protein of interest was detected by using mouse monoclonal anti-V5 tag antibody (1/2000; LifeTechnologies), rabbit polyclonal anti-endothelin receptor type B antibody (1/2000; Proteintech), rabbit monoclonal anti-glyceraldehyde-3-phosphate dehydrogenase (GAPDH) antibody (1/10,000; Cell Signaling Technology), horseradish peroxidase (HRP)-conjugated goat anti-mouse or anti-rabbit IgG (Jackson Immunoresearch Laboratories), and Immobilon Western Chemiluminescent HRP Substrate (Millipore).

Total proteins separated with SDS-PAGE were visualized by Oriole Fluorescent Gel Stain solution (Bio-Rad Laboratories) according to the manufacturer’s instructions.

### Cloning of the P2RY2 Open Reading Frame and Mutagenesis.

Complementary DNA was synthesized from total RNA extracted from HEK293 cells with TRIzol Reagent (Molecular Research Center, Inc.) by using SuperScript IV (LifeTechnologies) and then amplified by KOD FX Neo DNA polymerase (TOYOBO). The PCR product encoding the open reading frame (ORF) of the wild-type human P2RY2 and EDNRB were cloned into the pEF6/V5-His-TOPO vector (LifeTechnologies). The P2RY2 ORF fused to the V5-His6 tag at the C terminus, and the EDNRB ORF without a tag were subcloned into the multicloning site of the pHAGE-CMV(CMV promoter)-MCS(multi-cloning site)-IZsGreen vector (EvNO00061605; the PlasmID Harvard Medical School) with the In-Fusion high-definition (HD) cloning kit (TaKaRa Bio). Based on the plasmid DNA (pDNA) encoding wild-type human P2RY2, mutant P2RY2s with V5-His6 tags at the C terminus (F114A and F261A) were generated by Q5 Site-Directed Mutagenesis Kit according to the manufacturer’s protocols. Primers used in the plasmid construction are described below.

The primer set used for cloning of wild-type human P2RY2 is as follows: forward primer, ATG GCA GCA GAC CTG GGC CCC TGG A; reverse primer, CAG CCG AAT GTC CTT AGT GTT CTC.

The primer set used for cloning of wild-type human EDNRB is as follows: forward primer, ATG CAG CCG CCT CCA AGT CTG, reverse primer; TCA AGA TGA GCT GTA TTT ATT.

Primer sets used for the mutagenesis of P2RY2 are as follows: F114A mutant forward primer, CCT CTT CTT CAC CAA CCT TTA CTG CAG; F114A mutant reverse primer, GTT GGT GAA GAA GAG GAA GCG CAC CAG; F261A mutant forward primer, CTG CCA GCT CAC GTC ACC CGC ACC C; and F261A mutant reverse primer, GAC GTG AGC TGG CAG GAA GCA GAG G. Underlined bases correspond to mutant codons in each primer sequence.

### Cloning of the P2RY4 and P2RY11 ORF.

For cloning of wild-type human P2RY4 and P2RY11, their entire ORF sequences fused to the V5-His6 tag at the C terminus, as in the case of P2RY2, were chemically synthesized by FASMAC Co. Ltd. and subcloned into the multicloning site of the pHAGE-CMV-MCS-IZsGreen vector with the In-Fusion HD cloning kit (TaKaRa Bio).

### Aequorin Cell-Based Assay.

The day before transfection, 293FT cells were seeded into a collagen-coated 10 cm dish and incubated at 37 °C in DMEM supplemented with 10% FBS, 100 U/mL penicillin, and 100 μg/mL streptomycin in a 5% CO_2_-humidified chamber. After 24 h incubation, eight micrograms of pDNA encoding Aequorin protein and one microgram of pDNA encoding P2RY2, P2RY4, P2RY11, and P2RY2 mutants were diluted with 2 mL of Opti-MEM (LifeTechnologies) and then mixed with 12.5 μL Plus Reagent (LifeTechnologies). As the other mixture, 500 μL Opti-MEM and 31.25 μL Lipofectamine LTX (LifeTechnologies) were mixed and incubated for 5 min at room temperature, and then the mixture was added to the diluent containing the plasmid DNA. After incubation for 30 min, the pDNA diluent mixed with the transfection reagent was added to cultured cells. The cells were trypsinized and seeded into a collagen-coated 96-well plate for four hours after incubation at 37° C. The day after seeding, coelenterazine h (Promega, Madison) diluted with HBSS/20 mM Hepes/0.1% BSA at a final concentration of 500 nM (80 μL for the agonist assay, and 70 μL for the antagonist assay) was added to each well after removing the culture medium. The cells were incubated at room temperature for four hours in the dark. After the incubation, agonist and antagonist assays described below were carried out.

A total of 20 μL agonist PSB1114, UTP, rATP, and aptamers were added to each well at the indicated concentrations by using the dispensing system of FlexStation 3 (Molecular Devices) and then examined for luminescent intensity with the device.

A total of 10 μL antagonist AR-C118925XX and aptamers were added to each well at the indicated concentrations. After that, 20 μL agonist PSB 1114 and UTP were added to each well at the indicated concentrations by using the dispensing system of FlexStation 3 (Molecular Devices), followed by measurement of the luminescent intensity with the device.

For the PAM assay to wild-type P2RY2, UTP and aptamers were mixed and added to each well at the indicated concentrations by using the dispensing system of FlexStation 3 and then examined for luminescent intensity with the device, as in the agonist assay.

### SELEX Library Construction.

Single-stranded DNA libraries were designed and purchased from GeneDesign Inc. The library sequences are as follows: B02-library, 5′- GTA CGC TAG GCG TTA GTC TC [N40] ATC GTA CGA CGG TCG TAC CC -3′ (where N40 stand for 40-nucleotide random sequence). For PCR amplification, reverse transcription, and in vitro transcription with T7 polymerase, primer sequences were designed and indicated as follows: B02-library forward primer, 5′-TAA TAC GAC TCA CTA TAG GGT ACG ACC GTC GTA CGA T-3′, B02-library reverse primer, 5′- GTA CGC TAG GCG TTA GTC TC-3′ (T7 promoter sequence is underlined).

### In Vitro Selection of Aptamer.

Aptamer selection was carried out basically according to a previous report (20). Detailed conditions were described in *SI Appendix*, Table S1. Briefly, the B02 library described above was amplified by PCR with ExTaq DNA polymerase (TaKaRa Bio). To preserve original sequences, PCR was limited to three cycles. The resultant double-stranded DNAs were subjected to in vitro transcription using 2′-fluoro-CTP, 2′-fluoro-UTP, 2′-deoxy-ATP, 2′-hydroxy-GTP (at final concentration of 2.5 mM) and Y639F mutant T7 RNA polymerase (at final concentration of 40 ng/μL). After an overnight incubation at 37 °C, single-stranded RNAs (ssRNAs) were purified with phenol/chloroform (Nacalai Tesque, Inc.) to remove proteins, and further purified with ultrafiltration column, Amicon Ultra 0.5 mL (molecular weight cutoff (MWCO) 30 kDa [Millipore]) to remove NTPs by centrifugation at 14,000 × *g* for 5 min. After discarding flow through, distilled water was added in the device up to 500 μL, and this washing procedure (centrifugation) was repeated by five times in total. The removal efficiency of NTPs is shown in *SI Appendix*, Fig. S15. Before selection, RNA pools were denatured at 95 °C for 5 min in 100 μL Systematic Evolution of Ligands by EXponential enrichment (SELEX) buffer (145 mM NaCl, 5.4 mM KCl, 0.8 mM MgCl_2_, 1.8 mM CaCl_2_, and 20 mM Tris HCl pH 7.6), followed by cooling on ice for 3 min. The refolded RNA pool and the VLP were mixed and incubated for 30 min with agitation at 1,500 rpm using PowerBLOCK Shaker (ATTO Corporation). After the first round, transfer RNA–blocking treatment to the target VLP–expressing P2RY2 and subtraction using the nontarget VLP–expressing EDNRB were carried out for reducing sequences bound to nonspecific and nontargeted proteins.

For separation of aptamers complexed with VLP from free RNA sequences, an ultrafiltration column, Vivaspin 500 with 100 K MWCO polyether sulfone (PES) (Sartrius), was used for the separation by centrifugation at 14,000 × *g* for 5 to 10 min. After discarding flow through, 500 μL of SELEX buffer was added and passed through the column by centrifugation several times as a washing process (*SI Appendix*, Table S1). The removal efficiency of free RNAs was confirmed and shown in *SI Appendix*, Fig. S2. Retained solution-containing aptamers complexed with VLP in the column were recovered and added to phenol/chloroform (Nacalai Tesque, Inc.) for extraction of bound RNA sequences. After ethanol precipitation with Dr. GenTLE Precipitation Carrier (TaKaRa Bio), all amount of the collected RNAs were subjected to reverse transcription with ThermoScript Reverse Transcriptase (LifeTechnologies) according to the manufacture’s protocols, and then the ssDNAs were subjected to PCR amplification with ExTaq DNA polymerase (TaKaRa Bio) until appropriate PCR cycles. The amplified double-stranded DNAs were transcribed with Y639F mutant T7 RNA polymerase and modified NTPs described above and in a previous report (20). At the final round, the enriched RNA library was mixed with not only target VLP (P2RY2) but also nontarget VLP (EDNRB) as positive and negative materials, respectively. Then, RNA sequences bound to target and nontarget VLP were subjected to HTS for efficient in silico analysis.

### HTS.

The sequencing procedure was carried out by means of the Ion PGM system with an Ion 314 chip according to the manufacture’s protocols (LifeTechnologies). The number of sequencing reads obtained from the target and nontarget VLPs were 240,644 and 208,455, respectively.

### Sequence Analysis.

Sequencing data were analyzed with FASTAptamer (21). Briefly, after trimming the accessory sequences such as a barcode, adaptor, and T7 promoter sequence, sequences coding aptamers were analyzed. Furthermore, sequences of less than 8 reads were removed in this analysis. Subsequently, cluster analysis was carried out with an edit distance set to six; thus, sequences possessing fewer than six base differences were assigned into an identical cluster. Then, the sequences with highest read number (or RPM) in each cluster were extracted as a representative sequence in each cluster. Furthermore, the candidates were narrowed down to sequences with more than 500 RPM in the positive data. Of those, sequences showing enrichment values 0.5 or less were identified as final candidates. Note that the enrichment value “y/x” 0.5 or less means here that the RPM value of the sequence was more than twice as enriched as in the positive data (set to “x”) obtained from target VLP (P2RY2) compared to the false-positive data (set to “y”) obtained from nontarget VLP (EDNRB).

To visualize the difference in affinity of the enriched library against VLP-expressing target and nontarget GPCR, a heatmap hierarchal clustering was carried out by using the above-mentioned analyzed data with FASTAptamer. Briefly, the RPM of each sequence in the data set obtained from the target or nontarget VLP groups was divided by the median of the RPM obtained from the target or nontarget VLP groups, respectively. The values of each sequence in the target or nontarget VLP were further converted to a logarithmic scale (the base two) and then subjected to a cluster analysis using Cluster 3.0 (http://bonsai.hgc.jp/∼mdehoon/software/cluster/software.htm) and TreeView (https://bitbucket.org/TreeView3Dev/treeview3/src/master/).

In order to identify promising candidates, software such as MEME (https://meme-suite.org/tools/meme) for discovery of motif, CLUSTALW (https://www.genome.jp/tools-bin/clustalw) for multiple sequence alignment, and Centroidfold (http://rtools.cbrc.jp/centroidfold/) for predicting the secondary structure of sequences, all of which are available from each web site, were used.

### Statistical Analysis.

Statistical analysis was performed by GraphPad Prism 8 (GraphPad Software). Curve fitting with nonlinear regression and the analysis of 50% effective concentration (EC_50_) and 50% inhibitory concetration (IC50) were also performed by the software. Except for the data in Fig. 4*A*, the obtained data were analyzed by one-way ANOVA followed by the Tukey’s multiple comparison. As for Fig. 4*A*, the data were analyzed by two-way ANOVA followed by the Tukey’s multiple comparison, whose result was shown in *SI Appendix*, Table S2. For all statistical analysis, the alpha value was set at 0.05.

## Supplementary Material

Supplementary File

Supplementary File

## Data Availability

All study data are included in the article and/or supporting information.
