# Supplementary material for: Nucleic acid ligands act as a PAM and agonist depending on the intrinsic ligand binding state of P2RY2
Source: Proc Natl Acad Sci U S A. 2021 Apr 28;118(18):e2019497118. doi: 10.1073/pnas.2019497118 (PMC8106294; doi:10.1073/pnas.2019497118)
Supplement: Supplementary File [file pnas.2019497118.sapp.pdf]

PNAS
www.pnas.org

**Supplementary Information for**

# Nucleic acid ligands act as a PAM and agonist depending on the intrinsic ligand binding state of P2RY2

Masaki Takahashi, Ryo Amano, Michiru Ozawa, Anna Martinez, Kazumasa Akita, Yoshikazu Nakamura

Corresponding authors: Masaki Takahashi, Yoshikazu Nakamura
Email: Masaki Takahashi, tmasaki@ims.u-tokyo.ac.jp, Yoshikazu Nakamura, nak@ims.u-tokyo.ac.jp

**This PDF file includes:**

Supplementary text (Supplementary Materials and Methods)
Figures S1 to S15
Tables S1 and S2

**Other supplementary materials for this manuscript include the following:**

Dataset S1

## Supplementary Materials and Methods

### Random RNA library as a control

As a control RNA sequence, we used chemically synthesized random RNA library (5'-GGG AGA ACT TCG ACC AGA AG [N35] TAT GTG CGC ATA CAT GGA TCC TC-3', where N35 stand for 35 nucleotide random sequence that are fully modified with 2' OMe).

### Fluorescent labeling of aptamers and cell counts with flow cytometry for calculation of the aptamers' dissociation constant

To estimate dissociation constant (*K*d value) of aptamer c37_7-40 and c37_40-74, these focused aptamers and random library were labeled with Alexa647 by using Ulysis Alexa 647 labeling kit (LifeTechnologies) according to the manufacturer's protocols. Before the assessment day, the pHAGE-P2RY2-IZsGreen vector, which encode human wild type P2RY2 tagged with IRES-GFP, was transfected into HEK293 cells with Lipofectamine2000 according to the manufacturer's protocols. Likewise, the pHAGE-EDNRB-IZsGreen vector was transfected into HEK293 cells as negative control cells. The day after transfection, various amount of each labeled RNA (at 2000, 1000, 500, 250, 125, 62.5, and 0 nM final concentration) were mixed with HEK293 cells over-expressing P2RY2 and EDNRB. The incubation buffer was SELEX buffer supplemented with 1mg/ml yeast tRNA and 1% BSA to reduce non-specific binding of the aptamers. After incubation for 20 min at room temperature, the cells were subjected to centrifugation at 800 x*g* for 5 min and then supernatant were removed. Collected cells were resuspended with SELEX buffer, and this washing process was repeated by three-times. After washing step, GFP- and Alexa647-double-positive cells were analyzed with BD FACSCant™ II flow cytometer (BD Biosciences, CA, USA). Briefly, the cells were counted until the number of cells (all events) reached 10,000 cells. Using pulse geometry gates (a height, wide, and area of forward scatter (FSC) and side scatter (SSC)), cell clumps were eliminated. After setting the pulse gates, a gate to select GFP-positive cells was further set for examining interaction of the aptamer with GFP-positive cells, which were expected to overexpress P2RY2 and EDNRB, and then, Alexa647(aptamer)-positive cells were counted. After curve fitting with nonlinear regression, *B*max and *K*d values of each RNA were analyzed by GraphPad Prism 8.

### Free RNA removal efficiency in washing process of VLP-aptamers complexes with ultrafiltration column

To achieve immobilization free separation method, we used an ultrafiltration column, Vivaspin 500 centrifugal concentrator MWCO 100kDa (Sartorius), to isolate aptamer

complexed with VLP from unbound RNAs. As a model sequence, tRNA from backer's yeast (Roche Diagnosis), whose lengths range from about 70 to 90 bases, was subjected to the evaluation of efficiency of washing process with the column because the length of typical RNA aptamer is close to that of tRNAs. We first prepared 20 μg / 20 μL and 10 μg / 20 μL tRNA solutions, which are corresponding to the amount of RNAs used in first and final rounds in our SELEX, respectively. Two micro liters of them were left for electrophoresis, and the remaining tRNAs were transferred into an ultrafiltration column device. After addition of SELEX buffer up to 500 μL, the diluted tRNAs was subjected to a centrifugation at 14,000 xg for 5 min. Of the retaining tRNA in the device, two micro liters of the tRNA was saved for electrophoresis, and then a series of dilution process was repeated by four-times (total five times). Saved tRNAs and DynaMarker® RNA Low II (BioDynamics Laboratory Inc. Tokyo, Japan) were mixed with two micro liters of formamide and one micro liter of 6x loading buffer, and then heated at 90 $^{o}$C for 3 min. After that, those samples and the loading marker were subjected to a denaturing urea polyacrylamide electrophoresis at 6%T. Separating tRNAs were visualized with high-sensitive dye, SYBR™ Gold Nucleic Acid Gel Stain (ThermoFisher). Band intensities of RNAs in each lane was examined by ImageJ software.

**NTPs removal efficiency with ultrafiltration column in aptamer purification process**

To estimate the degree of carryover of NTPs from purified aptamers after IVT (*in vitro* transcription) to the incubation between aptamers and VLPs, we first prepared NTPs mixture at a final concentration of 2.5 mM in total 100 μL without aptamer library, which are same concentration and volume in the purification step of aptamers after IVT. Two micro liters of them were left for absorbance measurement, and the remaining NTP solution was transferred into an ultrafiltration column device. After addition of 400 μL of SELEX buffer, the diluted NTPs was subjected to a centrifugation at 14,000 xg for 5 min. Of the retaining NTPs in the device, two micro liters of the NTPs were saved for absorbance measurement, and then a series of dilution process was repeated by four-times (total five-times). Absorbance of the solution including NTPs was examined with NanoDrop™ 2000 (Thermo Scientific).

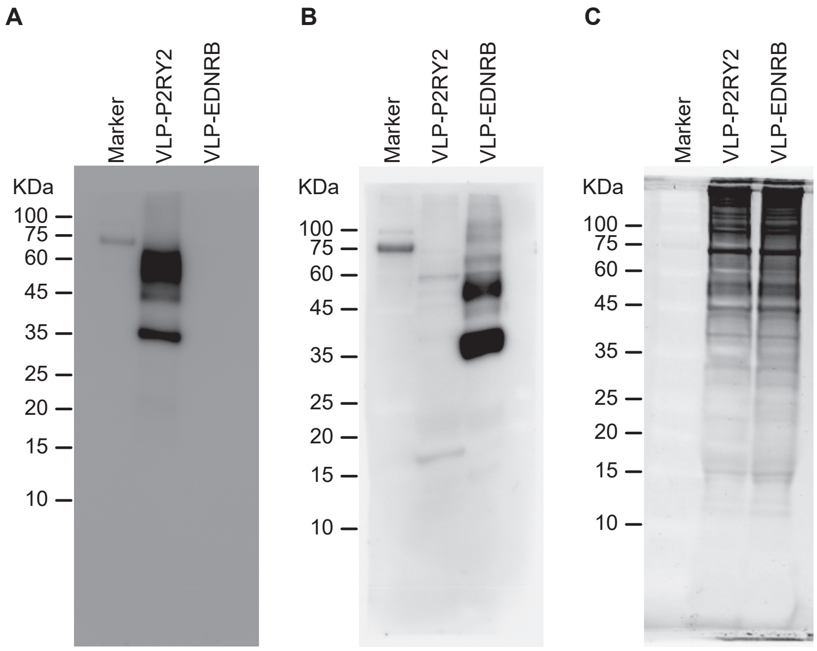


**Fig. S1. Expression of the target GPCR on VLPs.**

(**A**) Expression of P2RY2 on VLP. Total proteins of VLPs were subjected to western blotting. The human purinergic receptor P2Y2 (P2RY2) tagged with His6-V5 at N-terminal was detected by anti-V5 antibody. (**B**) Expression of EDNRB on VLP. As in (**A**), the human endothelin receptor type B (EDNRB) without tag was detected by anti-EDNRB antibody. (**C**) Total proteins as a loading control. Total proteins separated with SDS-PAGE were visualized by Oriole Fluorescent Gel Stain, showing no apparent changes between VLPs.

A

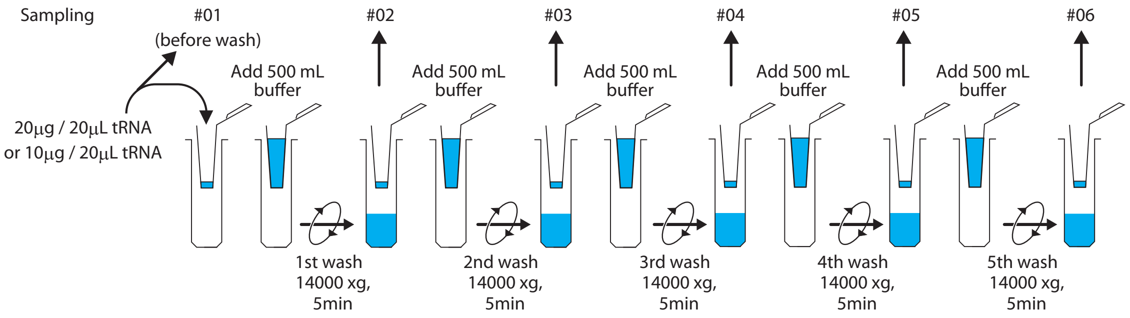


B

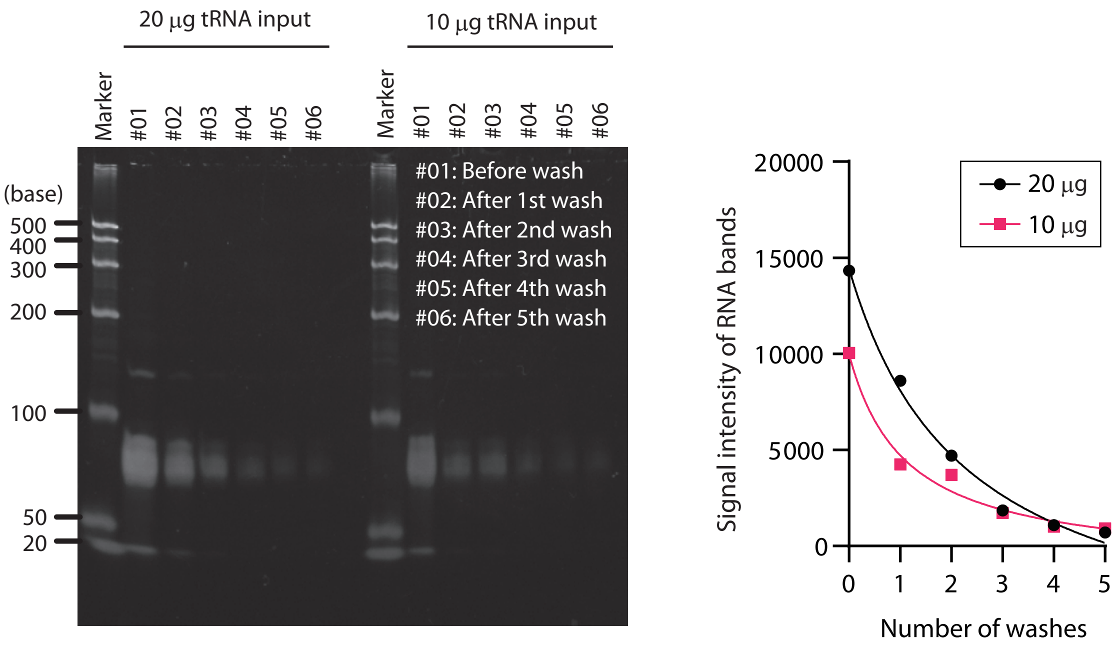


**Fig. S2. Free RNA removal efficiency in washing process of VLP-aptamers complexes with ultrafiltration column.** (**A**) Schematic drawing of performed experiment. To estimate the removal efficiency of free RNA sequences with ultrafiltration column (Vivaspin 500, MWCO 100K), approximately 20 and 10 µg of yeast tRNAs were subjected to the same washing procedure as the washing process in SELEX (details were described in Supplementary Materials and Methods). The washing procedure was repeated by five-times, and a portion of each remaining solution in the devices was subjected to denatured PAGE. (**B**) Visualization of RNAs with fluorescent dye on the gel. The separated tRNAs on the gel were visualized with high-sensitive dye, SYBR Gold gel staining dye. Washings three-times and five-times resulted in the reduction of 85 to 90% and 90 to 95% tRNAs, respectively.

A

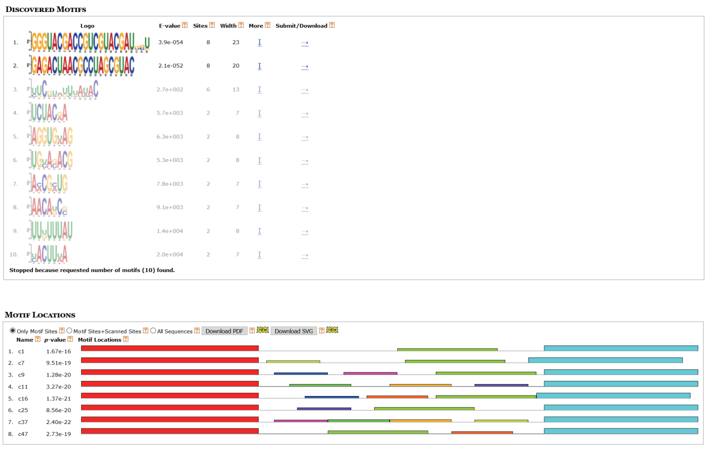


C

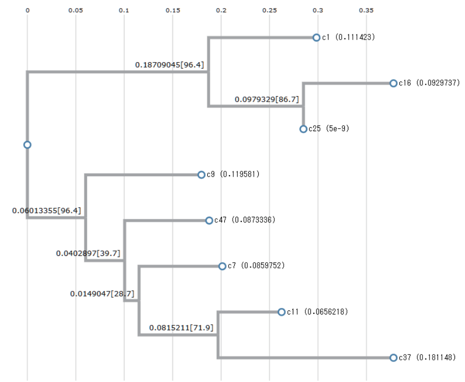


B

| Aptamer | Motif (5' to 3') | | | |
|---|---|---|---|---|
| c1 | A | | | |
| c7 | A | B | | |
| c9 | C | D | A | |
| c11 | E | F | G | |
| c16 | C | H | A | |
| c25 | G | A | | |
| c37 | D | E | F | B |
| c47 | A | H | | |

D

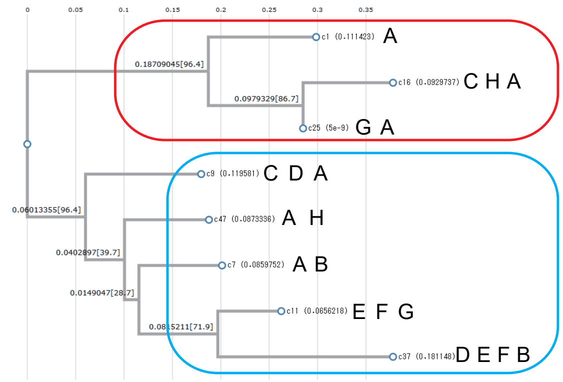


**Fig. S3. Sequence analysis based on the primary sequences of aptamers.** (**A**) Motif analysis. The eight sequences selected by the FASTAptamer were further subjected to motif analysis with MEME program. Except for the primer sequences, eight motifs were identified. (**B**) Motif compositions in selected aptamers. Eight motifs are denoted by alphabet from "A" to "H" . (**C**) Cluster analysis. The eight sequences were analyzed by the phylogenetic analysis with CLUSTALW program. (**D**) Summary of the motif and cluster analysis. The extracted motifs in (**A**, **B**) are described on the result of the phylogenetic analysis in (**C**). Based on the results, the eight sequences are divided into two major clusters, which are marked in red and blue lines. Because of the highest read number, c1 sequence was selected as candidate that represent a major cluster marked in red line. Subsequently, c11 and c37 were selected as candidates in the other major cluster marked in blue line, because the cluster analysis showed that c11 and c37 is located at the farthest position from c1 sequence. Besides, the sequences c11 and c37 relatively contained many motifs, which was not contained the motif "A" that are involved in all other sequences. Thus, c1, c11, and c37 sequences were selected as candidates and further subjected to the cell-based analysis.

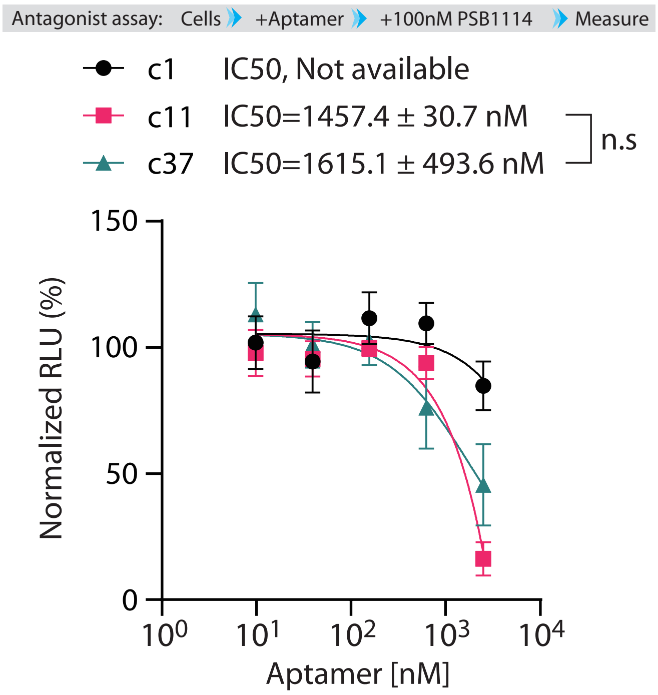


**Fig. S4. Antagonist assay of aptamers from first screening.** The antagonist activity of aptamers c1, c11, and c37, which were selected from first screening, was examined in the presence of PSB1114. Data represent the mean ± s.d. of three measurements. The values are expressed as RLU (in %) to the 100 nM PSB1114 level without aptamer after subtraction of basal LU in control cells without treatment. IC50 values of aptamers c11 and c37 were calculated and their differences were analyzed by t-test. n.s.: not significant.

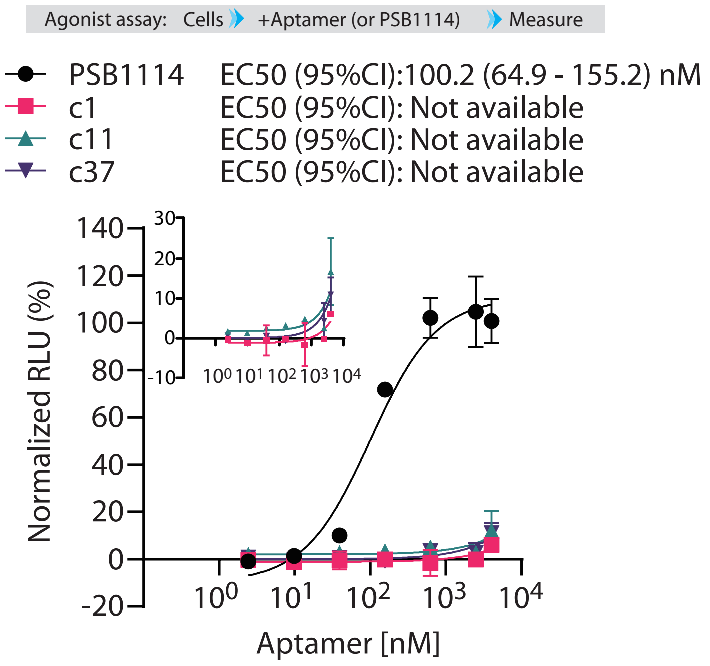


**Fig. S5. Agonist assay of aptamers from first screening.** The agonist activity of aptamers c1, c11, and c37, which were selected from first screening, was examined. Chemical agonist PSB1114 was used as a positive control. The aptamers and chemical agonist exposed to the cells overexpressing P2RY2 at final concentrations of 0, 2.44, 9.77, 39.1, 156.3, 625, 2500, and 4000 nM. The inset is an enlarged view focusing on the aptamers' agonistic activity, which seems not to reach a plateau even at 4000 nM. Data represent the mean ± s.d. of three measurements. The values are expressed as RLU (in %) to the 4000 nM PSB1114 level after subtraction of basal LU in control cells without treatment. EC50 value and 95% CI (confidence internal) of PSB1114 were indicated, but that of the aptamers was unavailable due to their weak activity.

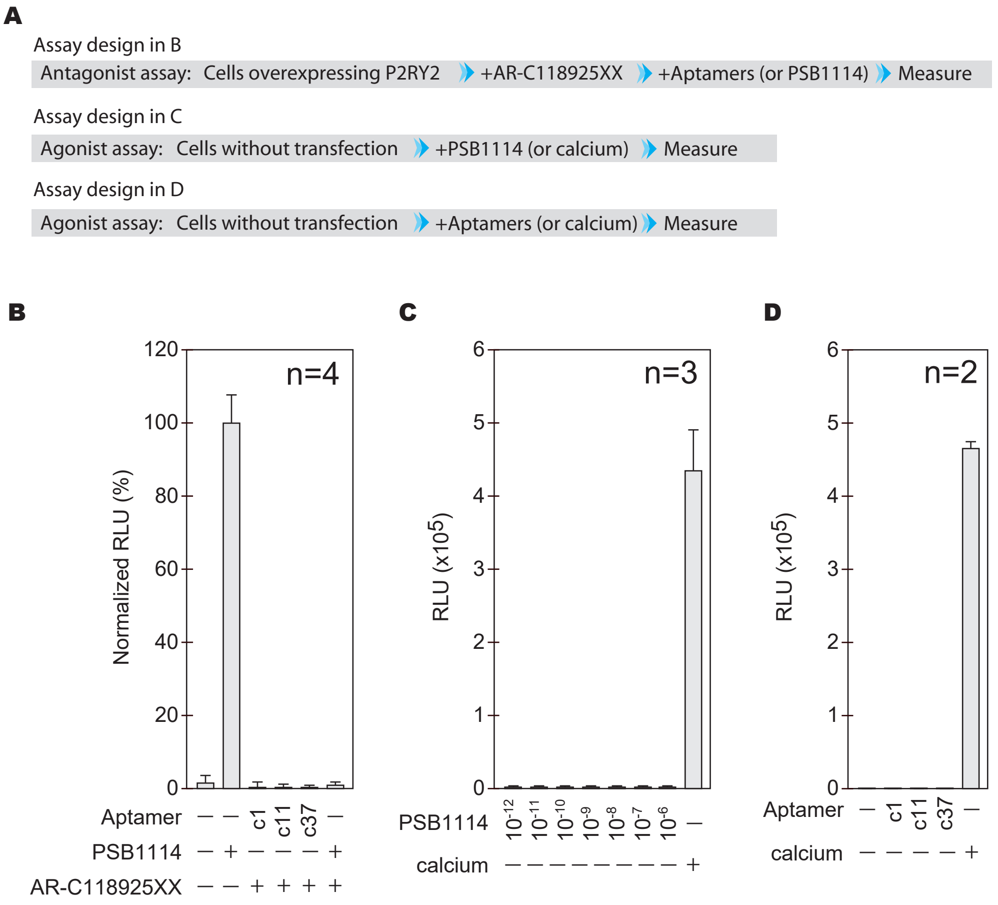


**Fig. S6. Target engagement of the aptamers.** (**A**) Assay designs for confirming target engagement of the aptamers. Schematic drawings of performed three experiments are shown, and their result are exhibited below as B, C, and D. (**B**) Inhibition of aptamer-induced P2RY2 activity by specific chemical antagonist. Target engagement of the aptamers against P2RY2 was confirmed by the pre-treatment of specific antagonist AR-C118925XX to the cells overexpressing P2RY2. The aptamers, AR-C118925XX, and PSB1114 (a positive agonist control) were used at final concentrations of 2.5, 10 μM, and 100 nM, respectively. Data represent the mean ± s.d. of four independent experiments. The values are expressed as RLU (in %) to the 100 nM PSB1114 level without aptamer after subtraction of basal LU in control cells without treatment. The data indicate that the aptamer-induced luminescent increase is mediated through P2RY2. (**C**) Calcium signaling via endogenous P2RY2. Without transfection of the P2RY2 expression plasmid, intact HEK293 cells were treated by PSB1114 at indicated concentrations, showing no response to even in the high dose treatment of PSB1114. Treatment of calcium at 200 mM was performed as a positive control. Data represent the mean ± s.d. of three independent experiments. The values were expressed as RLU. (**D**) Effect of aptamers on calcium signaling via endogenous P2RY2 in intact HEK293. Experimental procedures are the same as in (**C**). Data represent the mean ± s.d. of two independent experiments. The values were expressed as RLU. The data indicate that the agonistic activity of aptamers is not mediated through endogenous P2RY2.

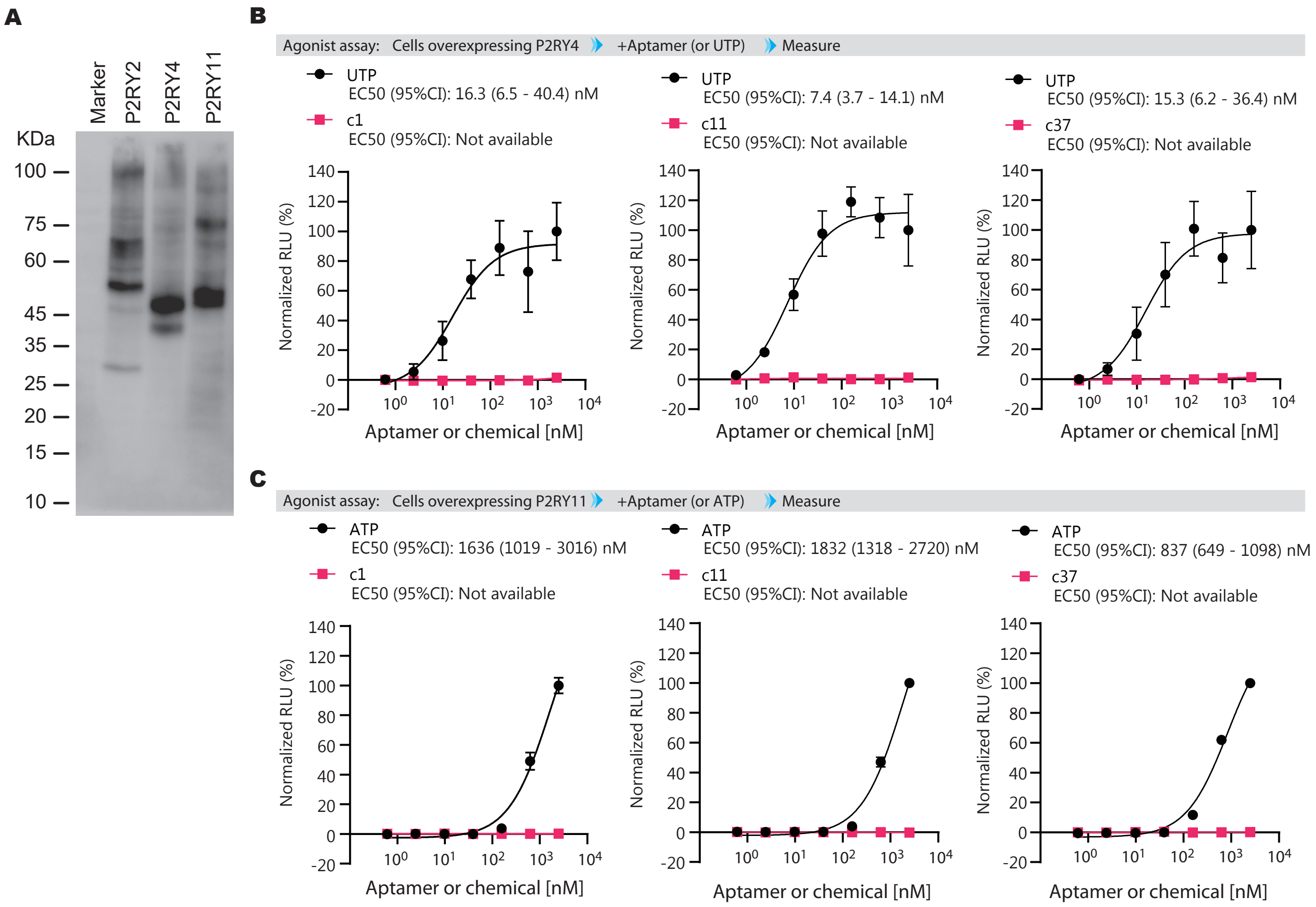


**Fig. S7. Agonistic activity of the aptamers to the P2Y family proteins.** (**A**) Expression of the P2Y family proteins. The expression levels of the P2Y family proteins P2RY4 and P2RY11 that are responsive to a common ligand with P2RY2, was confirmed by western blotting. Each expression plasmid encoding P2RY2, P2RY4 and P2RY11 ORF fused with the His6-V5 tag was transfected into HEK293 cells. Cell lysates were prepared 24 h after transfection, and the expression levels of each receptor was examined using anti-V5 antibody. (**B**) Agonist assay of the aptamers c1, c11 and c37 for P2RY4. Cells transfected with P2RY4 expression plasmid were exposed to the aptamers c1, c11, c37, and UTP (an endogenous agonist for P2RY4). (**C**) Agonist assay of the aptamers c1, c11 and c37 for P2RY11. Agonist assay as in (**B**) was carried out by using the aptamers c1, c11, c37, and ATP (an endogenous agonist). Data represent the mean ± s.d. of four independent experiments. The values are expressed as RLU (in %) to the 2.5 mM UTP/ATP level without aptamer after subtraction of basal LU in control cells without treatment. EC50 and 95% CI (confidence interval) of the aptamers and chemicals were shown when those values were available.

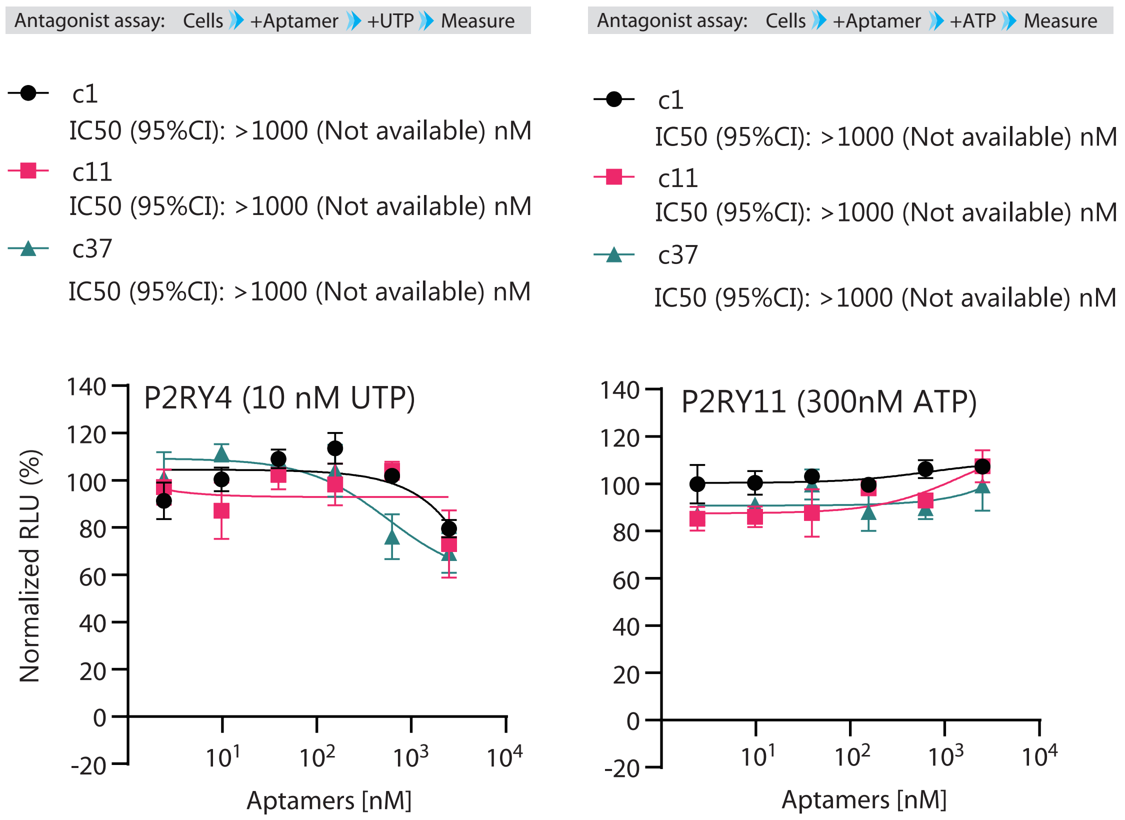


**Fig S8. Antagonist assay of aptamers to the P2Y family receptors.** Inhibitory activity of aptamers c1, c11, and c37 at the indicated concentrations to P2RY4 and P2RY11 was examined. Cells overexpressing P2RY4 and P2RY11 were post-treated by 10 nM UTP and 300 nM ATP, respectively. The values are expressed as RLU to the 10 nM UTP level for P2RY4 and the 300 nM ATP level for P2RY11 without aptamer. Data represent the mean ± s.d. of three independent experiments. The aptamers did not show obvious inhibitory activity to the family receptors. IC50 and 95% CI (confidence interval) values of each aptamer were calculated but they were unavailable due to their weak or no activity.

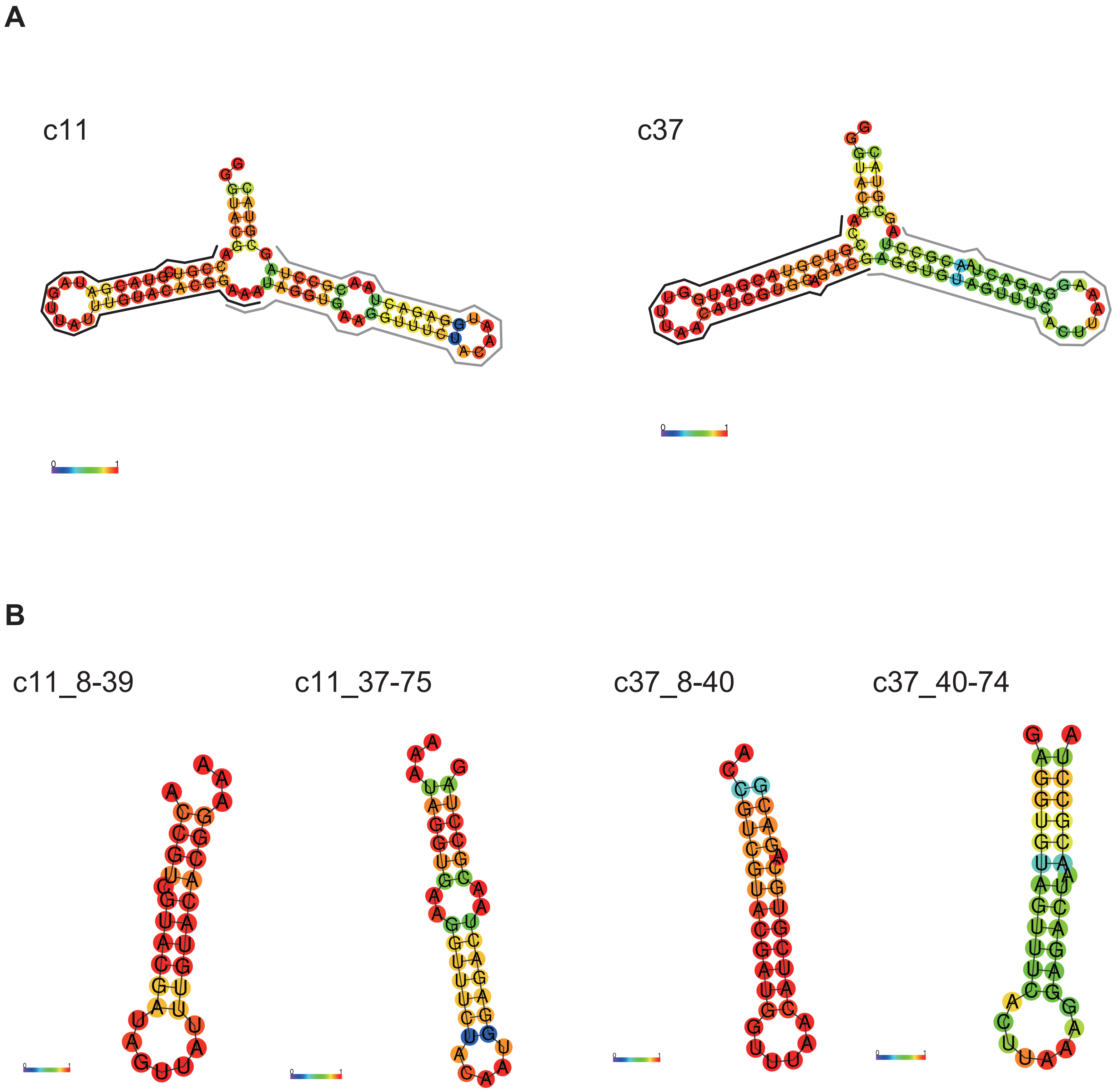


**Fig. S9. Predicted secondary structure of the full-length and truncated aptamers.** (**A**) Predicted secondary structure of the full-length aptamers. Secondary structure of the full-length aptamers c11 and c37 were predicted by Centroidfold. Based on these secondary structures, truncated aptamers were designed. The sequences along the lines in black and gray were synthesized as truncated aptamers. (**B**) Predicted secondary structure of the truncated aptamers. Secondary structure of the truncated aptamers was indicated as in (**A**). The c11 truncations c11_8-39 and c11_37-75 represent nucleotide positions 8 to 39 and 37 to 75 nucleotides, respectively. Likewise, the c37 truncations c37_8-40 and c37_40-74 represent nucleotide positions 8 to 40 and 40 to 74 nucleotides, respectively. The c37 full-length and its truncated aptamers are the same as shown in figure 3A.

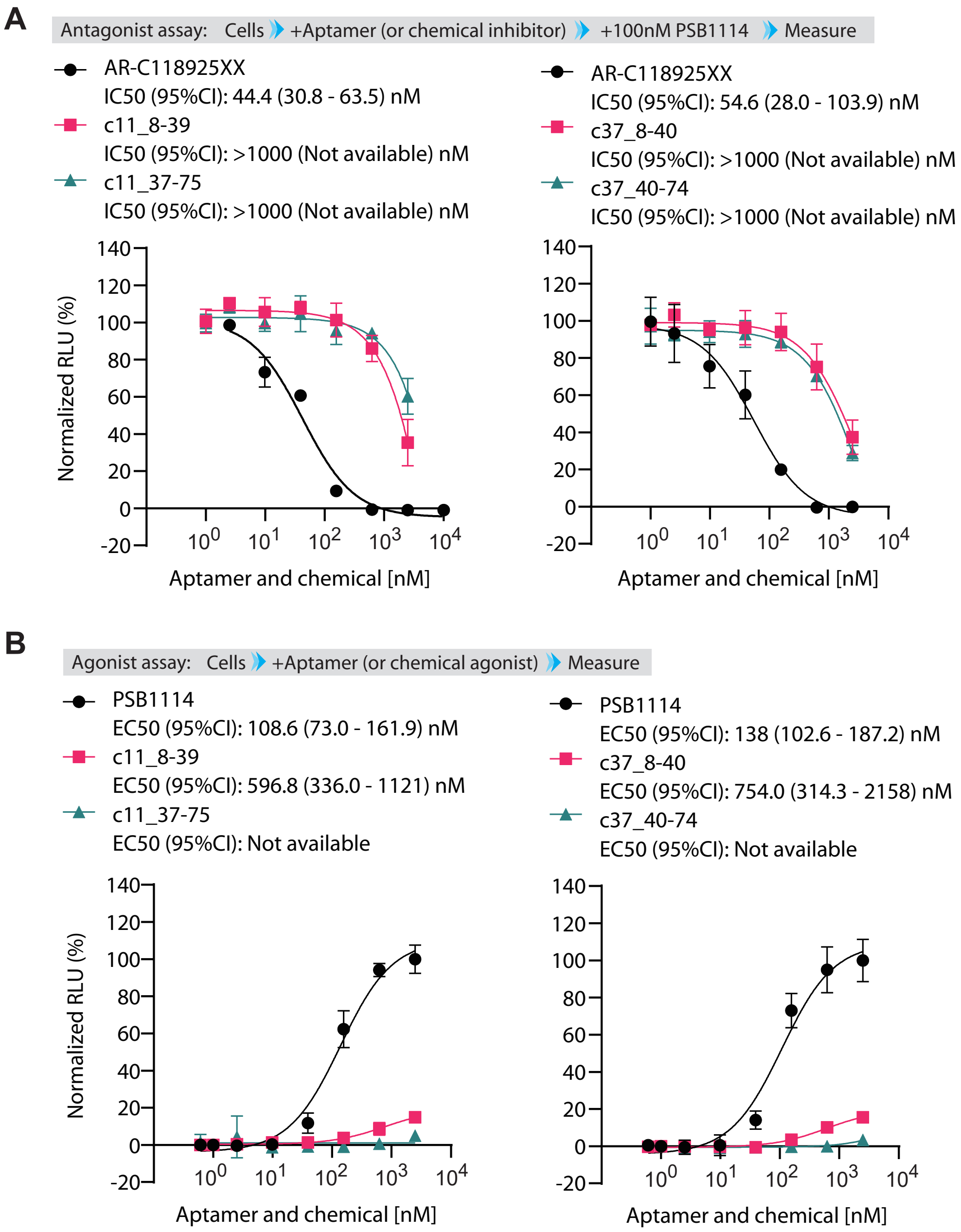


**Fig. S10. Agonist and antagonist assay of c11 and c37 truncated aptamers.** (**A**) Antagonist assay of the truncated aptamers. The inhibitory activity of four truncated aptamers (c11_8-39, c11_37-75, c37_8-40 and c37_40-74) was examined. Experimental conditions and results are the same as shown in Fig. 3B except for the addition of c11 truncated aptamers. (**B**) Agonist assay of the truncated aptamers. Data represent the mean ± s.d. of three independent experiments. The values are expressed as RLU (in %) to the 100 nM PSB level without aptamer after subtraction of basal LU in control cells without treatment. EC50, IC50, and 95% CI (confidence interval) of the aptamers and chemicals were shown when those values were available.

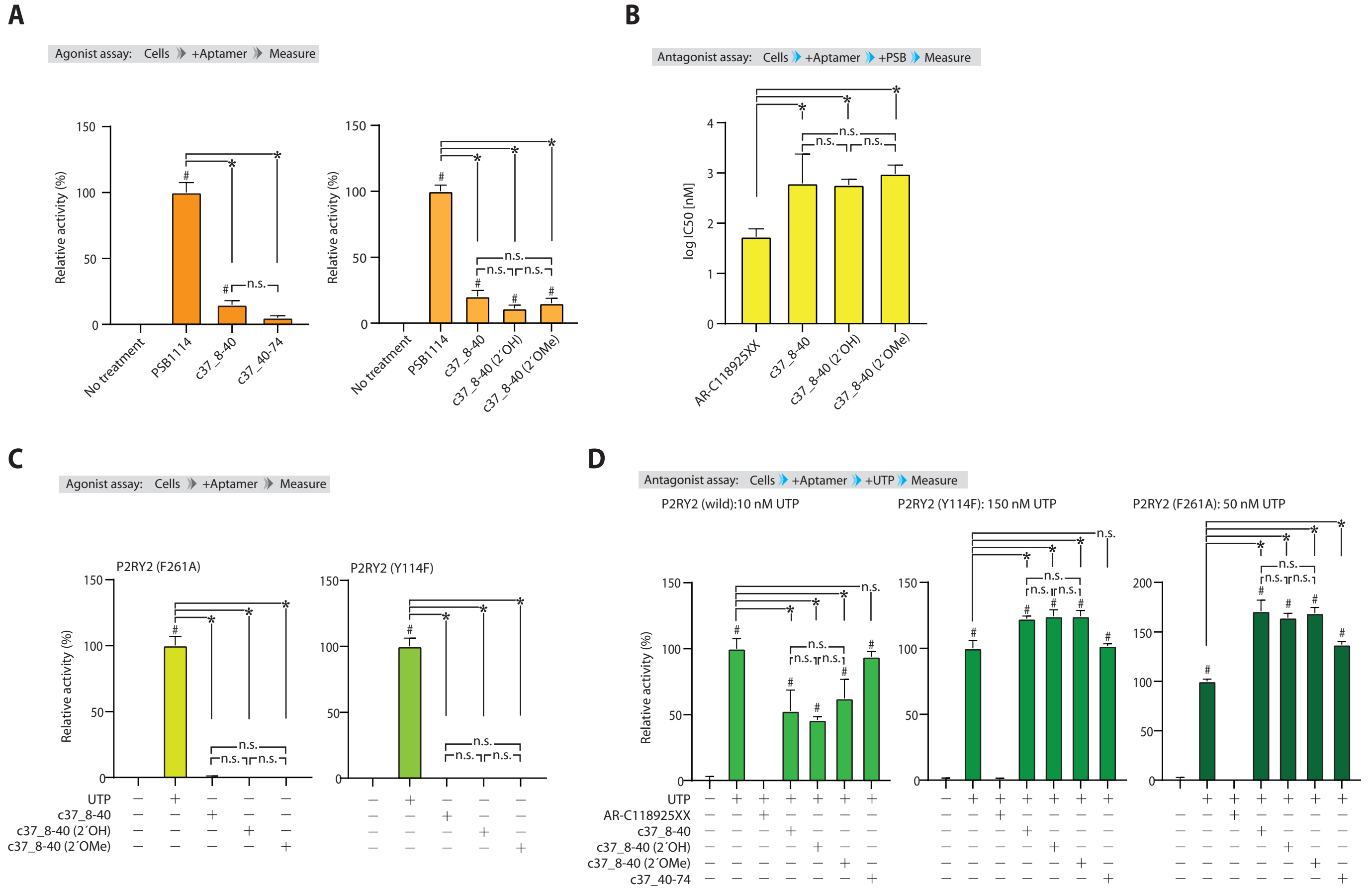


**Fig S11. Statistical analysis of the date in Fig.3.** Except for the data of antagonist assay in Fig.3B, the data obtained from 2.5 μM aptamer and chemical treatment in both agonist and antagonist assays were analyzed. (A) Statistical analysis of the date in Figs 3A and 3B. Statistical differences in the agonist activity of PSB1114, truncated (left panel) and modified aptamers (right panel) were analyzed. The values were expressed as RLU (in %) to the 2.5 μM PSB1114 level. (B) Statistical analysis of the date in Fig. 3B. Statistical difference in IC50 values of each aptamer and AR-C118925XX were analyzed when P2RY2-overexpressing cells were stimulated by post-treatment of 100 nM PSB1114. The values were expressed as RLU (in %) to the 100 nM PSB1114 level without any pre-treatment. (C) Statistical analysis of the date in Fig. 3C (left panel). As in (A), statistical differences in the agonist activity of UTP and indicated aptamers were analyzed. The values were expressed as RLU (in %) to the 2.5 μM UTP level. (D) Statistical analysis of the date in Fig. 3C (right panel). Statistical differences of the receptors' activities in the antagonist assay by using indicated aptamers and AR-C118925XX were analyzed when indicated P2Y2 receptors-overexpressing cells were stimulated by post-treatment of UTP (final 10, 150, 50 nM for wild, Y114F, and F261A mutant, respectively). The values were expressed as RLU (in %) to the 10 nM, 150 nM, 50 nM UTP level without aptamers for wild, Y114F, and F261A mutant receptor, respectively. Data represent the mean ± s.d of three independent experiments. All analyses were performed by one-way analysis of variance (ANOVA) followed by Tukey' s multiple tests. Sharp (#): *P*<0.05 versus no treatment group. Astarisk (*): *P*<0.05 between indicated groups. n.s.: not significant between indicated groups.

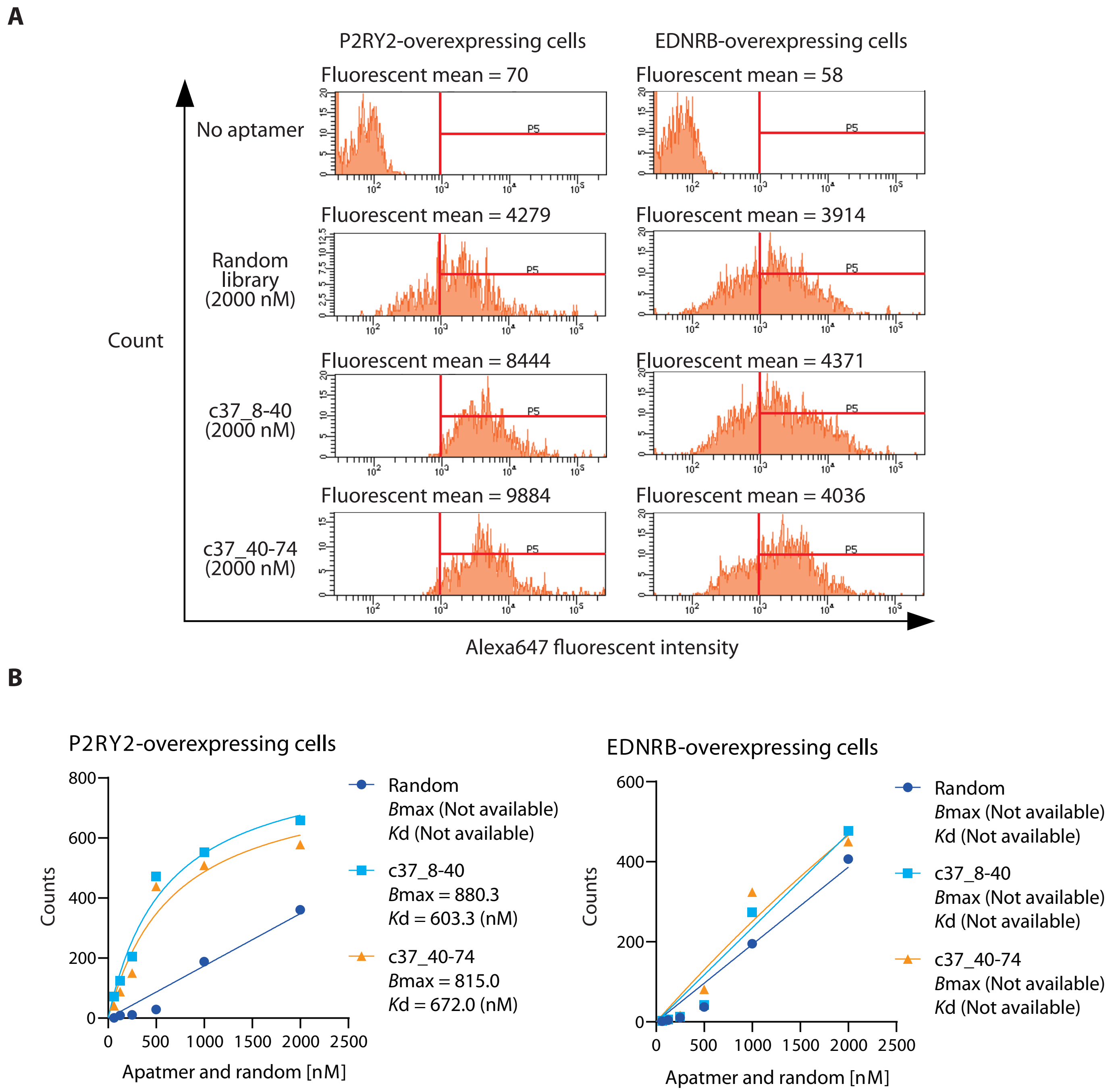


**Fig S12. Dissociation constant of truncated aptamers.** To examine binding affinity of aptamers, varied concentration of Alexa647-labeled aptamers c37_8-40, c37_40-74, and random RNA library were mixed with HEK293 cells overexpressing wild type P2RY2 and EDNRB (as a negative control), which were fused with IRES-GFP, and then the cells were examined by flow cytometry. (**A**) Flow cytometry histograms. After elimination of aggregated cells and GFP-negative cells by setting pulse gates and a GFP fluorescent parameter respectively, Alexa647-positive cells were examined by histograms when the Alexa647-labeled aptamers c37_8-40, c37_40-74, and random library at 2000 nM were mixed with P2RY2- and EDNRB-overexpressing cells. Fluorescent means of each histogram were indicated in the figure, which were calculated from entire histogram without any additional gate. The areas that were marked in red line were used for counting cell number of Alexa647-positive cells in the following figure to calculate each *K*d value of the aptamers. (**B**) Estimation of *K*d values of aptamers. After setting pulse gates to exclude aggregated cells and a gate for GFP fluorescent parameter to select P2RY2- and EDNRB-overexpressing cells, Alexa647-positive cells were counted in each sample that were mixed with various concentrations of the Alexa647-labeled aptamers and the cells overexpressing P2RY2 and EDNRB. After curve fitting with nonlinear regression, based on their *B*max values, the dissociation constant (*K*d) of each aptamer was estimated. Each dot represents single measurement.

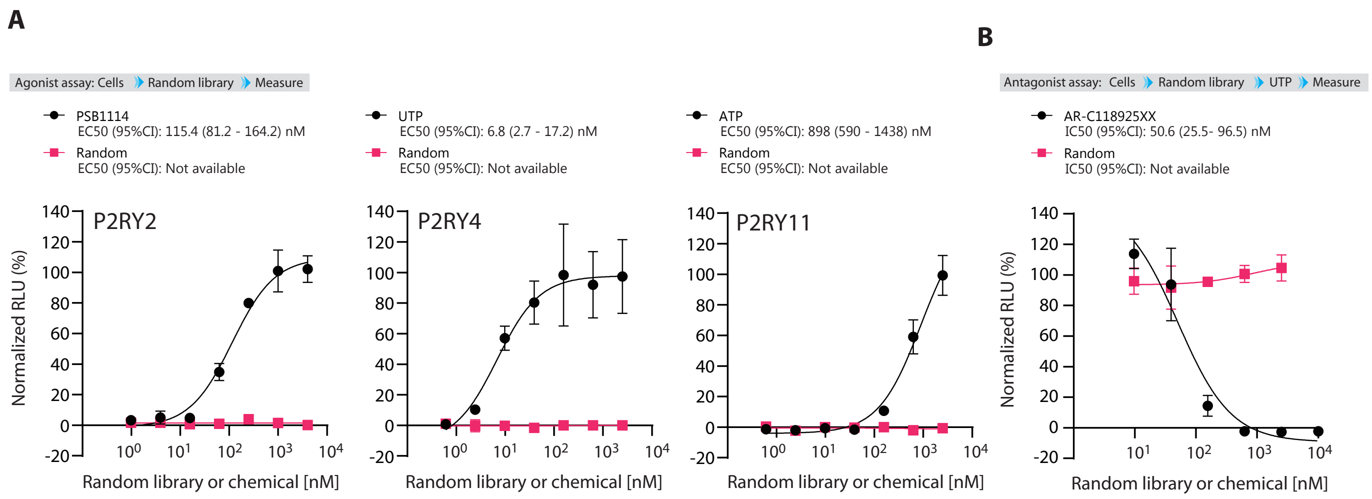


**Fig. S14. Agonist and antagonist assay of random RNA library.** (A) Agonistic activity of random RNA library to P2Y receptors. Agonist activity of random RNA library at indicated concentrations was examined against P2RY2, P2RY4, and P2RY11. Various amount of PSB1114, UTP, and ATP were used as positive controls for P2RY2, P2RY4, and P2RY11, respectively. The values were expressed as RLU (in %) to the 2.5 μM PSB1114, UTP, and ATP level for P2RY2, P2RY4, and P2RY11, respectively. (B) Inhibitory effect of random RNA library on P2RY2 activity. Inhibitory activity of random RNA library at indicated concentrations was examined against P2RY2. Data represent the mean ± s.d. of three independent experiments. The values were expressed as RLU (in %) to the 10 nM UTP without aptamer. IC50, EC50 and 95% CI (confidence interval) values of the library and chemicals were shown when those values were available.

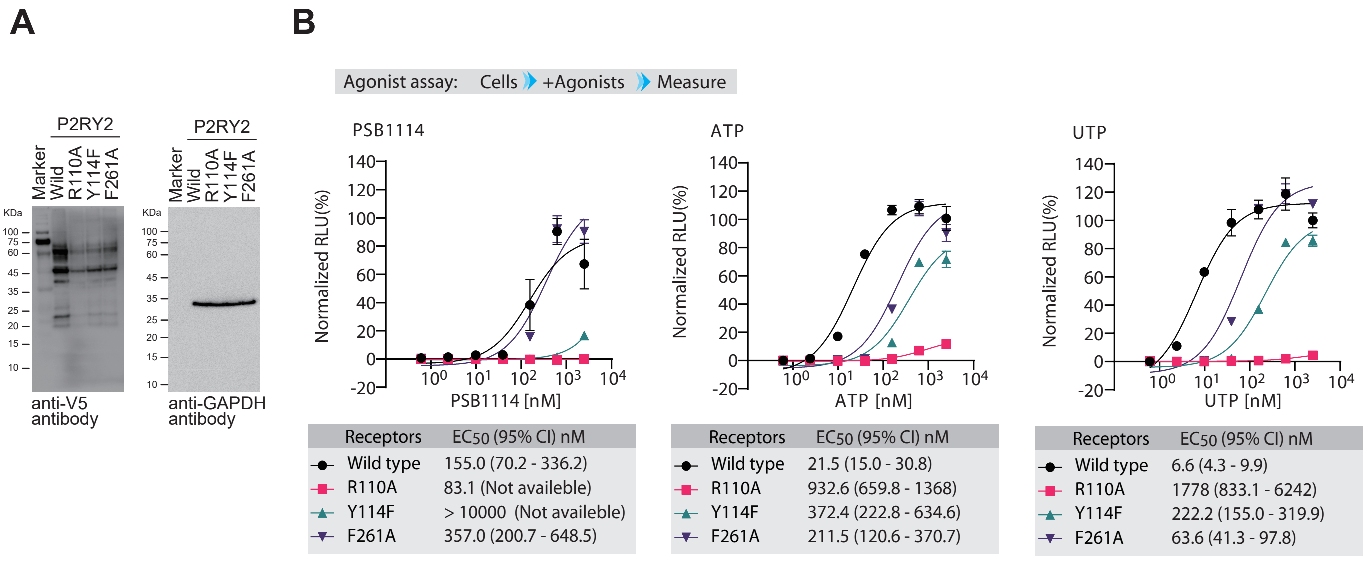


**Fig. S13. The response of the P2RY2 mutants to agonists.** (**A**) Expression of the mutant receptors. The expression plasmids encoding the wild, R110A, Y114F, and F261A mutant P2RY2, were transfected into HEK293 cells. The day after transfection, cell lysates were prepared and subjected to western blotting using indicated primary antibodies. (**B**) The response of the P2RY2 mutants, R110A, Y114F, and F261A, to agonists, UTP, rATP, and PSB1114. Because the R110A mutant showed weak or inactive to every agonist, the Y114F and F261A mutants were subjected to the further assessments. Data represent the mean ± s.d. of three independent experiments. The values are expressed as RLU (in %) to the 2.5 μM UTP level without aptamer after subtraction of basal LU in control cells without treatment. EC50 and 95% CI (confidence interval) of the chemical agonists against wild and mutant P2RY2s were shown when those values were available.

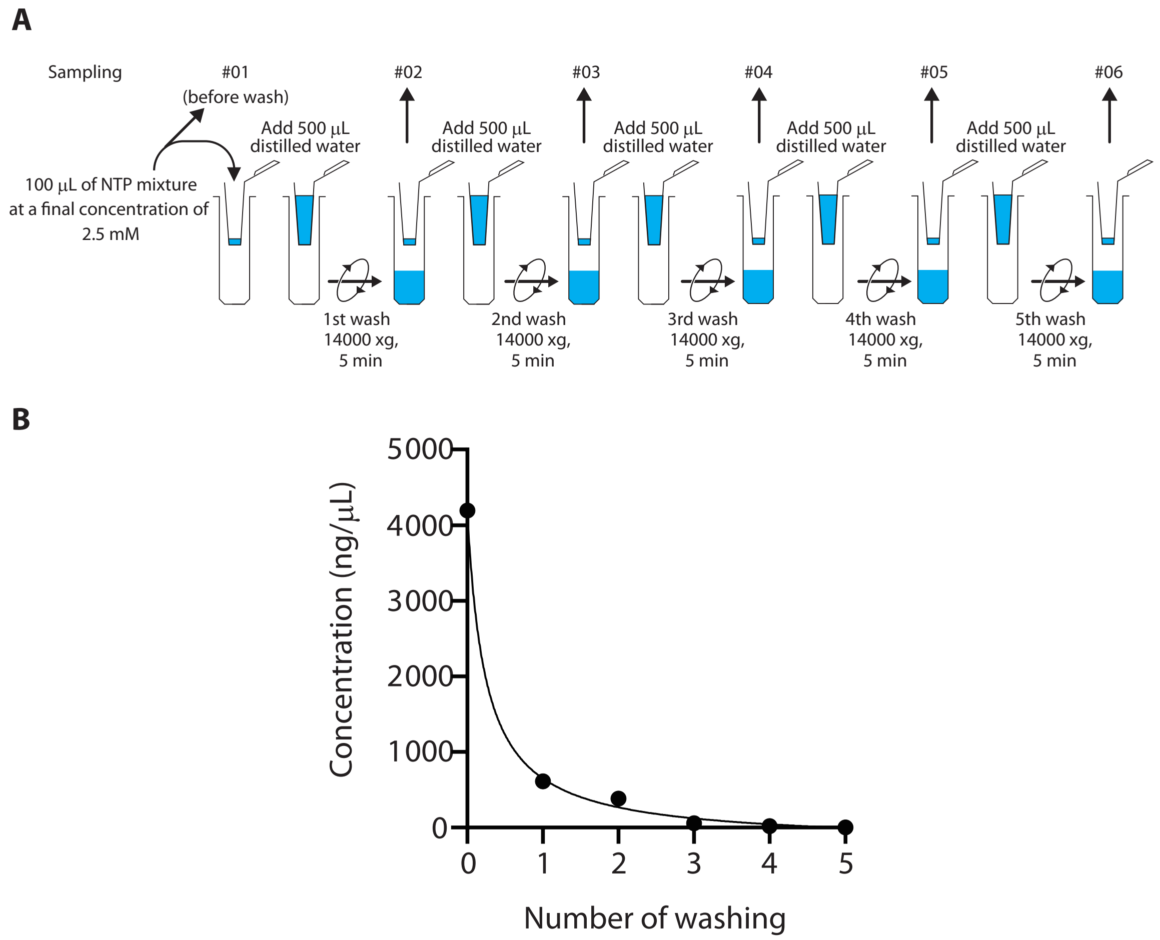


**Fig. S15. NTP removal efficiency in washing process with ultrafiltration column.** (**A**) Schematic drawing of performed experiment. To estimate the removal efficiency of NTPs from aptamer-synthetic process with ultrafiltration column (Amicon Ultra 0.5ML, MWCO 30K), NTPs at final concentration of 2.5 mM in 100 μL were subjected to the same washing procedure as the purification of aptamers in SELEX (details were described in Supplementary Materials and Methods). The process was repeated by 5-times, and a portion of each remaining solution in the devices was subjected to absorbance measurement. (**B**) Alterations of the concentration of NTPs in the washing process. Washings five-times resulted in the reduction of NTPs more than 99.9%.

Supplementary Table S1. SELEX condition in each round.

| Round # | 1R | 2R | 3R | 4R | 5R | 6R | 7R |
|---|---|---|---|---|---|---|---|
| RNA (micro gram) | 20 | 20 | 15 | 10 | 10 | 10 | 10 |
| VLPs vol. for subtraction (micro liter)*1 | none | 5 | 10 | 15 | 20 | 20 | 30 |
| tRNA (1mg/ml) for blocking (micro liter)*2 | none | 5uL to 5uL VLPs | 10uL to 5uL VLPs | 15uL to 5uL VLPs | 20uL to 4uL VLPs | 30uL to 3uL VLPs | 40uL to 2uL VLPs |
| VLPs vol. for selection (micro liter) | 5 | 5 | 5 | 5 | 4 | 3 | 2 |
| Incubation vol. (micro liter)*3 | 100 | 500 | 500 | 1000 | 1000 | 2000 | 2000 |
| Wash*4 | 3-times | 3-times | 3-times | 3-times | 4-times | 4-times | 5-times |

*1. VLP expressing EDNRB was used in the subtraction process to remove non-specifc and non-targeted RNA sequences.

*2. Transfer RNA (tRNA) was disolved in SELEX buffer and added to VLP expressing P2RY2 as a blocking agent. After addition of tRNA to the VLP suspension, incubation volume is adjusted with SELEX buffer.

*3. Incubation of the RNA pool with VLPs was performed at room temperature in the indicated volume. The volume was adjusted with SELEX buffer.

*4. Five hundred micro liter of SELEX buffer was added to ultrafiltration column and subjected to centrifugation to reduce volume, and then flow through were discarded. This procedure was repeated to remove free RNA sequences from the complexes as a washing process.

Table S2. Results of statistical analyses in the data of Fig.3A (right panel).

| Two-way ANOVA | | |
|---|---|---|
| Effect of UTP | F (3, 56) = 462.9 | P <0.0001 |
| Effect of Aptamer | F (6, 56) = 28.18 | P <0.0001 |
| Effect of UTP x Aptamer | F (18, 56) = 6.025 | P <0.0001 |

Tukey's multiple comparison

| Comparison | 95% CI | P value |
|---|---|---|
| UTP 0nM:No aptamer vs UTP 0nM:Aptamer 2.4nM | -31.52 ～ 32.58 | >0.9999 |
| UTP 0nM:No aptamer vs UTP 0nM:Aptamer 9.8nM | -35.16 ～ 28.95 | >0.9999 |
| UTP 0nM:No aptamer vs UTP 0nM:Aptamer 39.1nM | -37.42 ～ 26.68 | >0.9999 |
| UTP 0nM:No aptamer vs UTP 0nM:Aptamer 156.3nM | -37.24 ～ 26.86 | >0.9999 |
| UTP 0nM:No aptamer vs UTP 0nM:Aptamer 625nM | -41.29 ～ 22.81 | >0.9999 |
| UTP 0nM:No aptamer vs UTP 0nM:Aptamer 2500nM | -50.10 ～ 14.00 | 0.8969 |
| UTP 0nM:No aptamer vs UTP 5nM:No aptamer | -69.38 ～ -5.275 | 0.0074 |
| UTP 0nM:No aptamer vs UTP 5nM:Aptamer 2.4nM | -63.07 ～ 1.036 | 0.0701 |
| UTP 0nM:No aptamer vs UTP 5nM:Aptamer 9.8nM | -68.30 ～ -4.194 | 0.0112 |
| UTP 0nM:No aptamer vs UTP 5nM:Aptamer 39.1nM | -73.33 ～ -9.225 | 0.0015 |
| UTP 0nM:No aptamer vs UTP 5nM:Aptamer 156.3nM | -109.2 ～ -45.11 | <0.0001 |
| UTP 0nM:No aptamer vs UTP 5nM:Aptamer 625nM | -129.5 ～ -65.42 | <0.0001 |
| UTP 0nM:No aptamer vs UTP 5nM:Aptamer 2500nM | -127.4 ～ -63.32 | <0.0001 |
| UTP 0nM:No aptamer vs UTP 10nM:No aptamer | -86.42 ～ -22.32 | <0.0001 |
| UTP 0nM:No aptamer vs UTP 10nM:Aptamer 2.4nM | -82.74 ～ -18.63 | <0.0001 |
| UTP 0nM:No aptamer vs UTP 10nM:Aptamer 9.8nM | -93.96 ～ -29.86 | <0.0001 |
| UTP 0nM:No aptamer vs UTP 10nM:Aptamer 39.1nM | -102.9 ～ -38.77 | <0.0001 |
| UTP 0nM:No aptamer vs UTP 10nM:Aptamer 156.3nM | -125.2 ～ -61.12 | <0.0001 |
| UTP 0nM:No aptamer vs UTP 10nM:Aptamer 625nM | -133.7 ～ -69.61 | <0.0001 |
| UTP 0nM:No aptamer vs UTP 10nM:Aptamer 2500nM | -134.9 ～ -70.77 | <0.0001 |
| UTP 0nM:No aptamer vs UTP 100nM:No aptamer | -136.7 ～ -72.59 | <0.0001 |
| UTP 0nM:No aptamer vs UTP 100nM:Aptamer 2.4nM | -153.0 ～ -88.87 | <0.0001 |
| UTP 0nM:No aptamer vs UTP 100nM:Aptamer 9.8nM | -162.3 ～ -98.22 | <0.0001 |
| UTP 0nM:No aptamer vs UTP 100nM:Aptamer 39.1nM | -138.0 ～ -73.85 | <0.0001 |
| UTP 0nM:No aptamer vs UTP 100nM:Aptamer 156.3nM | -167.4 ～ -103.3 | <0.0001 |
| UTP 0nM:No aptamer vs UTP 100nM:Aptamer 625nM | -158.8 ～ -94.71 | <0.0001 |
| UTP 0nM:No aptamer vs UTP 100nM:Aptamer 2500nM | -146.1 ～ -81.99 | <0.0001 |
| UTP 5nM:No aptamer vs UTP 5nM:Aptamer 2.4nM | -25.74 ～ 38.36 | >0.9999 |
| UTP 5nM:No aptamer vs UTP 5nM:Aptamer 9.8nM | -30.97 ～ 33.13 | >0.9999 |
| UTP 5nM:No aptamer vs UTP 5nM:Aptamer 39.1nM | -36.00 ～ 28.10 | >0.9999 |
| UTP 5nM:No aptamer vs UTP 5nM:Aptamer 156.3nM | -71.88 ～ -7.779 | 0.0028 |
| UTP 5nM:No aptamer vs UTP 5nM:Aptamer 625nM | -92.20 ～ -28.10 | <0.0001 |
| UTP 5nM:No aptamer vs UTP 5nM:Aptamer 2500nM | -90.09 ～ -25.99 | <0.0001 |
| UTP 5nM:No aptamer vs UTP 10nM:No aptamer | -49.09 ～ 15.01 | 0.939 |
| UTP 5nM:No aptamer vs UTP 10nM:Aptamer 2.4nM | -45.41 ～ 18.69 | 0.9967 |
| UTP 5nM:No aptamer vs UTP 10nM:Aptamer 9.8nM | -56.63 ～ 7.469 | 0.3877 |
| UTP 5nM:No aptamer vs UTP 10nM:Aptamer 39.1nM | -65.55 ～ -1.446 | 0.0305 |
| UTP 5nM:No aptamer vs UTP 10nM:Aptamer 156.3nM | -87.90 ～ -23.80 | <0.0001 |
| UTP 5nM:No aptamer vs UTP 10nM:Aptamer 625nM | -96.39 ～ -32.29 | <0.0001 |
| UTP 5nM:No aptamer vs UTP 10nM:Aptamer 2500nM | -97.55 ～ -33.44 | <0.0001 |
| UTP 5nM:No aptamer vs UTP 100nM:No aptamer | -99.37 ～ -35.27 | <0.0001 |
| UTP 5nM:No aptamer vs UTP 100nM:Aptamer 2.4nM | -115.6 ～ -51.54 | <0.0001 |
| UTP 5nM:No aptamer vs UTP 100nM:Aptamer 9.8nM | -125.0 ～ -60.89 | <0.0001 |
| UTP 5nM:No aptamer vs UTP 100nM:Aptamer 39.1nM | -100.6 ～ -36.52 | <0.0001 |
| UTP 5nM:No aptamer vs UTP 100nM:Aptamer 156.3nM | -130.1 ～ -65.95 | <0.0001 |
| UTP 5nM:No aptamer vs UTP 100nM:Aptamer 625nM | -121.5 ～ -57.38 | <0.0001 |
| UTP 5nM:No aptamer vs UTP 100nM:Aptamer 2500nM | -108.8 ～ -44.67 | <0.0001 |
| UTP 10nM:No aptamer vs UTP 10nM:Aptamer 2.4nM | -28.37 ～ 35.73 | >0.9999 |
| UTP 10nM:No aptamer vs UTP 10nM:Aptamer 9.8nM | -39.59 ～ 24.51 | >0.9999 |
| UTP 10nM:No aptamer vs UTP 10nM:Aptamer 39.1nM | -48.51 ～ 15.59 | 0.957 |
| UTP 10nM:No aptamer vs UTP 10nM:Aptamer 156.3nM | -70.86 ～ -6.757 | 0.0041 |
| UTP 10nM:No aptamer vs UTP 10nM:Aptamer 625nM | -79.35 ～ -15.25 | 0.0001 |
| UTP 10nM:No aptamer vs UTP 10nM:Aptamer 2500nM | -80.50 ～ -16.40 | <0.0001 |
| UTP 10nM:No aptamer vs UTP 100nM:No aptamer | -82.33 ～ -18.23 | <0.0001 |
| UTP 10nM:No aptamer vs UTP 100nM:Aptamer 2.4nM | -98.61 ～ -34.50 | <0.0001 |
| UTP 10nM:No aptamer vs UTP 100nM:Aptamer 9.8nM | -108.0 ～ -43.85 | <0.0001 |
| UTP 10nM:No aptamer vs UTP 100nM:Aptamer 39.1nM | -83.59 ～ -19.48 | <0.0001 |
| UTP 10nM:No aptamer vs UTP 100nM:Aptamer 156.3nM | -113.0 ～ -48.91 | <0.0001 |
| UTP 10nM:No aptamer vs UTP 100nM:Aptamer 625nM | -104.4 ～ -40.34 | <0.0001 |
| UTP 10nM:No aptamer vs UTP 100nM:Aptamer 2500nM | -91.73 ～ -27.63 | <0.0001 |
| UTP 100nM:No aptamer vs UTP 100nM:Aptamer 2.4nM | -48.33 ～ 15.78 | 0.9617 |
| UTP 100nM:No aptamer vs UTP 100nM:Aptamer 9.8nM | -57.68 ～ 6.427 | 0.3097 |
| UTP 100nM:No aptamer vs UTP 100nM:Aptamer 39.1nM | -33.31 ～ 30.80 | >0.9999 |
| UTP 100nM:No aptamer vs UTP 100nM:Aptamer 156.3nM | -62.74 ～ 1.366 | 0.0778 |
| UTP 100nM:No aptamer vs UTP 100nM:Aptamer 625nM | -54.17 ～ 9.935 | 0.5986 |
| UTP 100nM:No aptamer vs UTP 100nM:Aptamer 2500nM | -41.45 ～ 22.65 | >0.9999 |
